# Efficacy of pulmonary surfactant with budesonide in premature infants: A systematic review and meta-analysis

**DOI:** 10.1371/journal.pone.0312561

**Published:** 2025-01-09

**Authors:** Nanthida Phattraprayoon, Bing Tan, Mingkwan Na Takuathung

**Affiliations:** 1 Princess Srisavangavadhana College of Medicine, Chulabhorn Royal Academy, Bangkok, Thailand; 2 Department of Pharmacy, Affiliated Hospital of Youjiang Medical University for Nationalities, Baise, Guangxi, China; 3 Department of Pharmacology, Faculty of Medicine, Chiang Mai University, Chiang Mai, Thailand; University Medical Centre Ljubljana (UMCL) / Faculty of Medicine, University Ljubljana (FM,UL), SLOVENIA

## Abstract

Pulmonary surfactant (PS) is one of the main treatment for neonates with respiratory distress syndrome (RDS). Budesonide has recently been studied as an additional treatment in such cases, but there is limited evidence supporting this. This study was implemented to determine the efficacy of PS combined with budesonide in premature infants. To achieve this, we conducted a systematic review and meta-analysis of randomized controlled trials by searching PubMed, Scopus, Embase, and the Cochrane Library from inception until July 12, 2024. We utilized a random-effects model to calculate the risk ratio and mean differences (MDs) with 95% confidence intervals (CIs) for the clinical outcomes of PS with budesonide versus PS alone. We used the GRADE approach to assess the quality of the evidence. We included 26 randomized controlled trials with a total of 2701 patients in the analysis. Treatments of PS with budesonide and PS alone were compared in all trials. PS with budesonide reduced bronchopulmonary dysplasia (BPD) incidence (risk ratio, 0.61; 95% CI, 0.51, 0.73), duration of mechanical or invasive mechanical ventilation (MD, −2.21 days; 95% CI, −2.72, −1.71), duration requiring oxygen (MD, −5.86 days; 95% CI, −8.44, −3.29), and hospitalization time (MD, −5.61 days; 95% CI, −8.65, −2.56). These results were based on low to very low evidence certainty. Only moderate-to-severe BPD or severe BPD showed a significant reduction when PS was used in conjunction with budesonide, a finding supported by moderate evidence certainty. Our study showed that the administration of PS with budesonide significantly improved respiratory outcomes, including the incidence of BPD, duration of mechanical or invasive mechanical ventilation, duration requiring oxygen, and hospitalization time in preterm infants, without short-term adverse drug events. However, the evidence certainty was mostly low to very low.

## Introduction

Preterm infants are particularly prone to respiratory problems and complications due to their undeveloped lungs [1, 2]. Some respiratory diseases can recover spontaneously, but such conditions may still have an impact on infants’ growth and development [1, 3]. Extremely premature newborns are at risk of respiratory distress syndrome (RDS) [1]. It is caused by low production of pulmonary surfactant (PS) [4, 5] which may result in bronchopulmonary dysplasia (BPD) [6].

Pulmonary surfactant is a complex protein–lipid mixture produced by type II alveolar cells. It reduces alveolar surface tension, improves gas exchange, and prevents alveolar collapse [7]. The lack of PS in neonates results in respiratory distress, alveolar damage, and cytokine release [8]. Although multiple factors are known to cause BPD, research has indicated that inflammation plays an important role in its pathogenesis [9, 10]. This suggests that the use of steroids to decrease lung inflammation could be beneficial against BPD. Indeed, steroids have been introduced for premature infants with RDS or prolonged ventilation to help prevent further complications such as BPD [11]. Steroids can be administered systemically or via inhalation [12], with the former of these being particularly beneficial for treating these patients. Evidence has shown that systemic steroids can facilitate the successful extubation of patients [13] and reduce the rates of mortality and BPD at 36 weeks [14].

However, concerns have been raised regarding the systemic side effects associated with the use of systemic steroids, such as dexamethasone. It was, therefore, hypothesized that local steroid introduction to the lung would enhance efficacy and minimize adverse effects [15]. Budesonide, a locally high potency corticosteroid, is considered a supplementary treatment to be used in conjunction with PS for this vulnerable population. It is anticipated that the anti-inflammatory properties of steroids could reduce the respiratory morbidity associated with BPD and the severity of the condition [10]. The use of budesonide to supplement treatment with PS in infants with RDS to improve respiratory outcomes has been extensively explored [16, 17]. The study showed that administering budesonide with PS improved the rate of newborn BPD-free survival [17]. However, not all studies show a significant improvement in respiratory outcomes [18]. Despite the common use of dexamethasone and hydrocortisone systemically in preterm infants for the treatment and prevention of BPD, a thorough research has also investigated the potential benefit of local steroids in this regard. Certain side effects of inhaled corticosteroids, such as oral candida and dysphonia [19], are a limitation of using this type of steroids.

In our study, we focused on the effects of budesonide. This is particularly warranted, as conflicting results on the benefits of budesonide have been reported in terms of whether it is efficacious in conjunction with PS or can be administered using PS as a vehicle in the first few days of life in preterm infants [18]. Therefore, our systematic review and meta-analysis were performed to evaluate whether budesonide and PS affect respiratory and other clinical outcomes in preterm infants. We hypothesized that budesonide with PS improves preterm outcomes.

## Materials and methods

### Study protocol, registration, and ethical approval

We conducted a systematic review and meta-analysis following the Preferred Reporting Items for Systematic Reviews and Meta-Analyses (PRISMA). We registered the research protocol (CRD42023438989) with PROSPERO. This research has received a certificate of exemption from the Research Ethics Committee of the Princess Srisavangavadhana College of Medicine, Chulabhorn Royal Academy (No.055/2566).

### Data sources and search strategy

A comprehensive and methodical search was conducted on PubMed (National Library of Medicine, Bethesda, MD, USA), Scopus, Embase, and the Cochrane Library from their inception until July 12, 2024. The search terms were (“preterm” OR “premature”) AND [“surfactant” OR (“pulmonary surfactant”) OR (“exogenous surfactant”)] AND “budesonide.” The references of the identified papers were reviewed to find additional relevant studies.

### Eligibility criteria

The review included randomized controlled trials (RCTs) and human studies comparing PS plus budesonide to PS alone in preterm infants. There were no restrictions on the language in which the report was published.

### Study selection

Two researchers (NP and MN) reviewed the scientific literature separately and independently by reviewing the relevance, study design, methods, and outcomes in accordance with the inclusion criteria. Conflicts were resolved through discussions with the third researcher (BT).

### Data extraction and quality assessment

The reports on all included studies contained the following information: the first author’s name, study year, study design, participants’ country of origin, intervention type, and respiratory outcomes, including PS administrations, ventilation duration, and BPD incidence. Data on other preterm complications and side effects of corticosteroid were also extracted. When data were missing or unreported, or if we required additional data, we sent enquiries to the researchers via email. Bias was investigated using the revised Cochrane Risk of Bias Assessment for Randomized Trials. This classification defines studies as having low or high bias or having some concerns about bias based on the RoB2 algorithm. The RoB2 assessment results were visually represented using robvis.

### Data synthesis and statistical analysis

We used a random-effects model to compute risk ratios (RRs) for categorical variables and weighted mean differences (MDs) for continuous variables. We provided a 95% confidence interval (CI) for each estimate. An intervention-based subgroup analysis was used to identify the source of heterogeneity. In Q-statistic and heterogeneity assessments, values of 25%, 50%, and 75% were considered to represent low, moderate, and high heterogeneity, respectively. A funnel plot was used to assess publication bias. P-values of < 0.05 were considered statistically significant. RevMan software was used for all meta-analyses.

The GRADE method with degradation criteria was used to categorize each outcome’s evidentiary certainty as high, moderate, low, or very low [20, 21]. This classification incorporated the risk of bias [22], inconsistency [23], indirectness [24], imprecision [25], and publication bias [26]. A GRADE evidence profile table was generated using the GRADEpro Guideline Development Tool (http://gradepro.org).

### Definitions

Respiratory distress syndrome was diagnosed using radiographic and clinical evidence. Bronchopulmonary dysplasia was defined for infants with respiratory distress at birth who required oxygen (> 21%) at a postmenstrual age of 36 weeks or > 28 days old. Mental Development Index (MDI) and Psychomotor Development Index (PDI) scores of ≤ 69 were considered to represent developmental delay [15, 27].

## Results

### Search results

**Fig 1:**
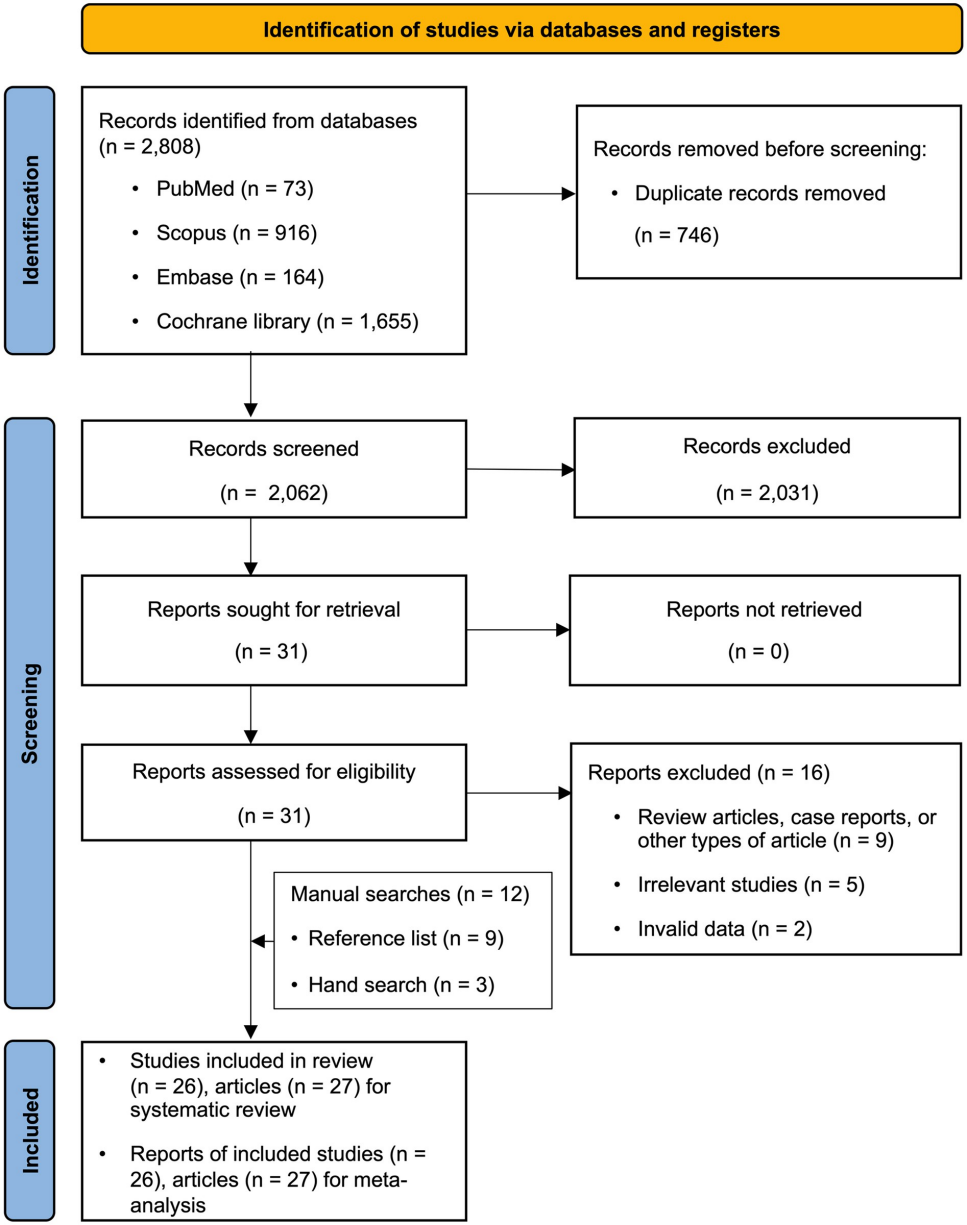
PRISMA flow diagram. Selection of studies for systematic review and meta-analysis.

The database search yielded 2808 citations. After evaluating the titles and abstracts, we reviewed the full text of 31 articles. Fourteen studies (15 articles) met our inclusion and meta-analysis criteria. Twelve additional publications were retrieved from the reference lists of the included studies and other sources. A total of 16 studies were excluded from the analysis (S5 Table), of which nine were non-randomized controlled trials, five were irrelevant, and two contained invalid data (Fig 1).

### Study characteristics

A meta-analysis of 26 RCTs (27 articles) [15–18, 27–49] with 2701 subjects was conducted. Among the subjects, 1343 received PS with budesonide, while 1358 received PS alone. Each clinical trial compared PS with budesonide to PS alone. In 19 RCTs [15–18, 28, 29, 31, 32, 37, 38, 40–44, 46–49], budesonide was administered by intratracheal (ITT) instillation with PS via an endotracheal tube or thin catheter, which was compared to PS alone administered by the same procedure. Meanwhile, in five RCTs, budesonide nebulization or inhalation with PS ITT was compared to PS ITT alone [33–36, 39]. In addition, one study [30] compared the use of nebulized PS and budesonide to nebulized PS alone. Finally, one study [45] evaluated PS alone (administration route and dosage unidentified) compared with nebulized or inhaled budesonide with PS (same as the control). Table 1 and S1 Table summarize the characteristics of the studies and participants. Overall, 19 trials were conducted in China, 1 in Taiwan [16, 27], and 5 in Iran [18, 33, 43, 48, 49]. One multicenter trial was conducted in China, Taiwan, and the United States [15]. The reports on these studies were published between 2008 and 2024. In total, 16 studies (17 articles) were reported in 2008–2019, while 10 were reported in 2020–2024.

**Table 1:** Characteristics of the included studies.

Study	Type of study	Location	Inclusion criteria	Exclusion criteria	Randomizationmethod	Study period	Comparator	N comparator	Control	N control
**Yeh 2008**	RCT	Taiwan	• BW < 1500 g • Severe RDS • Required MV with FiO_2_ ≥ 0.6 shortly after birth	• Severe congenital anomalies • Lethal cardiopulmonary disorder	Computer with permuted blocks	Sep 2004–Feb 2006	PS 100 mg/kg ITT with budesonide 0.25 mg/kg ITT q 8 h	60	PS 100 mg/kg ITT q 8 h	56
**Kuo 2010**	RCT (follow-up study)	Taiwan	• BW < 1500 g • Severe RDS • Required MV with FiO_2_ ≥ 0.6 shortly after birth • Survivor at 2 years old	• Severe congenital anomalies • Lethal cardiopulmonary disorder	Computer with permuted blocks	Sep 2004–Feb 2006	PS 100 mg/kg ITT with budesonide 0.25 mg/kg ITT q 8 h	35	PS 100 mg/kg ITT q 8 h	32
**Wan 2010**	RCT	China	• Preterm • RDS	NR	NR	Dec 2006–Feb 2009	PS 100 mg/kg ITT with budesonide 0.25 mg/kg ITT	31	PS 100 mg/kg ITT	31
**Ke 2016**	RCT	China	• GA < 32 wks • BW < 1500 g • RDS required MV or CPAP with FiO_2_ ≥ 0.6	• Severe congenital anomalies • Severe CHD	NR	Mar 2012–Sep 2014	PS 200 mg/kg ITT with budesonide 0.25 mg/kg ITT	46	PS 200 mg/kg ITT	46
**Yeh 2016**	RCT	Taiwan, China, and USA	• BW < 1500 g • Severe RDS (grades III–IV) required MV with FiO_2_ ≥ 0.5	• Severe congenital anomalies • Lethal cardiopulmonary disorder	Computer with permuted blocks	Apr 2009–Mar 2013	PS 100 mg/kg ITT with Pulmicort 0.25 mg/kg ITT	131	PS 100 mg/kg ITT	134
**Pan 2017**	RCT	China	• GA < 32 wks • BW < 1500 g • RDS grades III–IV • Intrauterine infection	• Severe CHD • Congenital anomalies • CDH • Respiratory malformations • Chromosomal abnormalities	NR	Jun 2015–Mar 2016	PS 70 mg/kg ITT with budesonide 0.25 mg/kg ITT	15	PS 70 mg/kg ITT	15
**Cao 2018**	RCT	China	• GA < 32 wks • RDS • Required MV	• Asphyxia • Severe congenital cardiopulmonary abnormalities	Random number table	Jan 2014–Feb 2015	PS 100 mg/kg inhalation with budesonide 0.25 mg/kg inhalation	40	PS 100 mg/kg inhalation	40
**Deng 2018**	RCT	China	• BW < 1500 g • AGA • RDS grades III–IV	• CHD • Congenital anomalies • CNS abnormalities • Severe congenital genetic metabolic disease • Pulmonary hemorrhage • ICH • Shock • Age > 8 h on admission • Mortality during treatment	NR	Jan 2014–Dec 2015	PS 150 mg/kg ITT with budesonide 0.25 mg/kg ITT	18	PS 150 mg/kg ITT	28
**Luo 2018**	RCT	China	• GA ≤ 32 wks • BW ≤ 1500 g • RDS • On MV or noninvasive respirator	• Severe CHD • Severe congenital anomalies • Shock • ICH • Sepsis	NR	Aug 2016–Aug 2017	PS 100 mg ITT with budesonide 0.25 mg ITT	75	PS 100 mg ITT	75
**Sadeghnia 2018**	RCT	Iran	• GA 23–28 wks • RDS • Required CPAP with FiO_2_ > 0.4	• Congenital malformations • Prenatal asphyxia	Odd/even document numbers	Jun 2014–Apr 2016	PS 100 mg/kg ITT (LISA) with budesonide 0.5 mg q 12 h NB (max. 7 days)	35	PS 100 mg/kg ITT (LISA)	35
**Wang 2018**	RCT	China	• RDS • Required MV	• CHD • Lung malformation	Random number table	Feb 2016– Aug 2017	PS 100 mg/kg ITT with budesonide 0.25 mg/kg inhalation (continuous for 3 days) for budesonide	72	PS 100 mg/kg ITT	72
**Yu 2018**	RCT	China	• RDS	• CHD • Congenital lung malformations • Severe systemic diseases	Random number table	Jan 2015– May 2017	PS 70 mg/kg ITT with budesonide 0.2–0.3 mg/kg inhalation (suspension) giving budesonide for 7 days	18	1) PS 70 mg/kg ITT (with NSS as a placebo)	16
**Du 2019**	RCT	China	• GA ≤ 32 wks • BW ≤ 1500 g • RDS	• Written informed consent not provided	NR	Oct 2013–Feb 2015	PS 100 mg/kg ITT with budesonide 0.25 mg/kg inhalation	30	PS 100 mg/kg ITT	30
**Ping 2019**	RCT	China	• GA ≤ 34 wks • BW < 1500 g • Severe RDS • Required MV	• Respiratory malformation • Air leak syndrome • Infection • Paralysis of respiratory muscle • CHD • Genetic metabolic disease • CNS abnormalities • Incomplete clinical data	Random number table	Aug 2015–Dec 2017	PS 150 mg/kg ITT with budesonide 0.25 mg/kg ITT	64	PS 150 mg/kg ITT	64
**Su 2019**	RCT	China	• GA < 28 wks • Required FiO_2_ > 0.3	• Major congenital anomalies • Severe uterine infection	Random number table	Dec 2016–Feb 2018	PS 200 mg/kg ITT with budesonide 0.25 mg/kg ITT	48	PS 200 mg/kg ITT	50
**Wang 2019**	RCT	China	• GA ≤ 32 wks • BW < 1500 g • RDS grades III–IV • Required invasive MV	• Severe congenital malformations • Inherited metabolic disease • Abnormalities of the CNS • ICH • Massive pulmonary hemorrhage • Shock • GBS infection	Random number table	Dec 2016–Feb 2018	PS 70 mg/kg ITT with budesonide 0.5 mg inhalation	28	PS 70 mg/kg ITT	28
**Zhou 2019**	RCT	China	• GA < 37 wks • BW < 1500 g • Severe RDS	• Congenital respiratory tract malformations • Respiratory muscle paralysis • GBS infection • Air leak syndrome • Aspiration pneumonia • Congenital genetic metabolic disease • CHD • Shock • Severe neurological abnormalities	Random number table	Aug 2015– Dec 2017	PS 150 mg/kg ITT with budesonide 0.25 mg/kg ITT	55	PS 150 mg/kg ITT	55
**Chen 2020**	RCT	China	• GA ≤ 32 wks • BW ≤ 1500 g • RDS	NR	NR	Dec 2017–Jan 2018	PS 100 mg/kg ITT with budesonide 0.25 mg/kg ITT	30	PS 100 mg/kg ITT	30
**Chuanlong 2021**	RCT	China	• GA < 35 wks • BW < 2500 g • RDS	NR	NR	Jan 2017– Jun 2019	PS 70 mg/kg ITT with budesonide 0.25 mg/kg ITT	39	PS 70 mg/kg ITT	39
**Gharehbaghi 2021**	RCT	Iran	• GA < 30 wks • BW < 1250 g • RDS • Required FiO_2_ > 40% • Required surfactant	• Major congenital anomalies • Birth asphyxia (Apgar score < 4 at 5 min after birth) • Lethal cardiopulmonary disorder	Block randomization	Oct 2017–Jun 2018	PS 200 mg/kg ITT with budesonide 0.25 mg/kg ITT	64	PS 200 mg/kg ITT	64
**Yang 2021**	RCT	China	• GA < 33 wks • BW ≤ 1500 g • RDS • Needed surfactant • Required respiratory support with FiO_2_ > 0.3	• Severe congenital abnormalities • Fatal cardiopulmonary diseases • Complex congenital heart diseases, • Congenital malformations in the respiratory system	Random number table	Oct 2016–Oct 2019	PS 70–140 mg ITT with budesonide 0.25 mg/kg ITT (if BW < 1.4 kg, 70 mg given; if BW ≥ 1.4 kg, 140 mg given ITT)	97	PS 70–140 mg ITT (if BW < 1.4 kg, 70 mg given; if BW ≥ 1.4 kg, 140 mg given)	101
**Yao 2021**	RCT	China	• GA ≥ 28 but < 32 weeks • BW < 1500 g • Required MV	• Hormone therapy within 28 days after birth • Congenital heart disease • Severe infectious diseases • Liver and kidney failure • Pneumothorax • Pulmonary infection	Random number table	Dec 2017– Dec 2019	PS, dosage, and route not reported (same as for control) with budesonide 0.5 mL inhalation twice a day for 2 wks	47	PS, dosage and route not reported	47
**Zheng 2021**	RCT	China	RDS	• Asphyxia • CHD • Genetic disorders • Moderate-to-severe bronchiectasis	Random number table	Jun 2018–Dec 2019	PS 100 mg/kg ITT with budesonide 0.25 mg/kg ITT	43	PS 100 mg/kg ITT	43
**Liu 2022**	RCT	China	• GA < 32 wks • BW < 1500 g • RDS • Respiratory support and PS required in 4 h after birth	• Severe congenital malformations • Fatal cardiopulmonary diseases • Congenital genetic metabolic diseases and immunodeficiency diseases • Early i.v. use of glucocorticoids due to hypotension or hypoglycemia	Random number table	Jan 2021– Jul 2021	PS 200 mg/kg ITT with budesonide 0.25 mg/kg ITT	60	PS 200 mg/kg ITT	62
**Armanian 2023**	RCT	Iran	• GA < 30 wks • Required NCPAP with FiO_2_ ≥ 30%	• Congenital malformation • Asphyxia • Sepsis	Permuted block randomization of size 6	Mar 2020– Oct 2021	PS 200 mg/kg 100 mg/kg for subsequent doses ITT if required, with budesonide once 0.25 mg/kg ITT	95	PS 200 mg/kg ITT	95
**Safa 2023**	RCT	Iran	• GA < 37 wks • BW 800–1500 g • Moderate-to-severe RDS • Required mechanical ventilation	• Severe congenital anomalies • Lethal cardiopulmonary disorder • Asphyxia • Sepsis	NR	Dec 2020–Jan 2022	PS 200 mg/kg ITT, with budesonide 0.25 mg/kg ITT	35	PS 200 mg/kg ITT	35
**Marzban 2024**	RCT	Iran	• GA < 37 wks • BW > 700 g • RDS • GA < 37 wks and BW < 2500 g, with requirement for mechanical ventilation within 4 h after birth • GA > 28 wks with requirement for FiO_2_ > 40% • GA < 28 wks with requirement for FiO_2_ > 30%	• BW < 700 g • Severe congenital anomalies • Fatal cardiopulmonary disease • Other causes of respiratory distress, such as CDH	Covariate adaptive randomization	2021	PS 200 mg/kg ITT, with budesonide 0.25 mg/kg ITT	67	PS 200 mg/kg ITT	67

**Abbreviations:** BW: birth weight; CDH: congenital diaphragmatic hernia; CHD: congenital heart disease; CNS: central nervous system; CPAP: continuous positive airway pressure; g: gram; FiO_2_: fraction of inspired oxygen; GA: gestational age; GBS: Group B *Streptococcus*; h: hour; ICH: intracranial hemorrhage; ITT: intratracheal; IV: intravenous; LISA: less invasive surfactant administration; MV: mechanical ventilation; NB: nebulization; NCPAP: nasal continuous positive airway pressure; NR: not reported; PS: pulmonary surfactant; RDS: respiratory distress syndrome; RCT: randomized controlled trial; wk: week

### Risk of bias assessment

Six studies (seven articles) [15, 16, 18, 27, 30, 48, 49], in which the randomization processes were described and defined as having a low risk. Two studies involved deviations from the intended interventions. Loss to follow-up and selective reporting were adequate. According to the revised Cochrane Risk of Bias Assessment, 5 studies had a low risk of bias, 1 had a high risk, and 20 had some concerns regarding risk of bias (S1 Fig).

### Efficacy of PS with budesonide compared to PS alone on clinical outcomes

**Fig 2:**
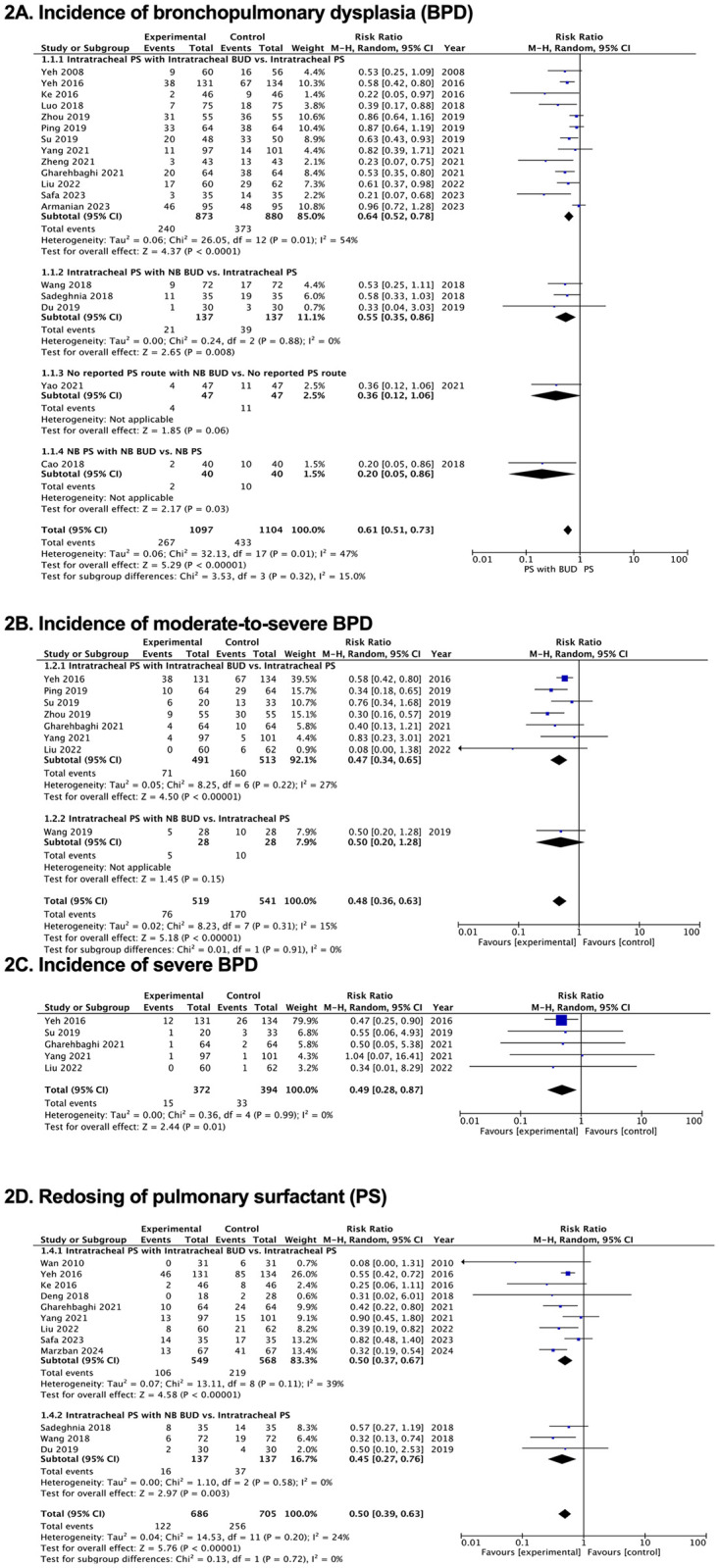
Forest plots of effects of pulmonary surfactant with budesonide on clinical outcomes in neonates. (A) Incidence of bronchopulmonary dysplasia (BPD). (B) Incidence of moderate-to-severe BPD. (C) Incidence of severe BPD. (D) Redosing of pulmonary surfactant (PS). BUD, budesonide; NB, nebulization; PS, pulmonary surfactant.

**Fig 3:**
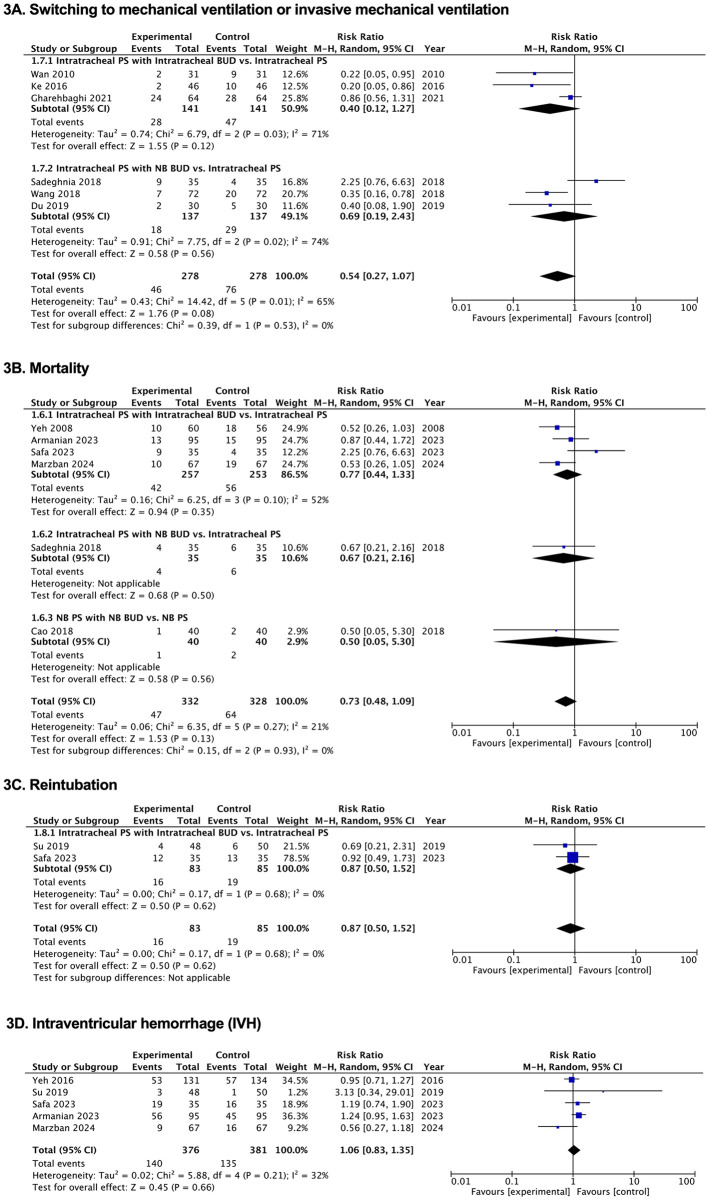
Forest plots of the effects of pulmonary surfactant with budesonide on clinical outcomes in neonates. (A) Switching to mechanical ventilation or invasive mechanical ventilation. (B) Mortality. (C) Reintubation. (D) Intraventricular hemorrhage (IVH). BUD, budesonide; NB, nebulization; PS, pulmonary surfactant.

**Fig 4:**
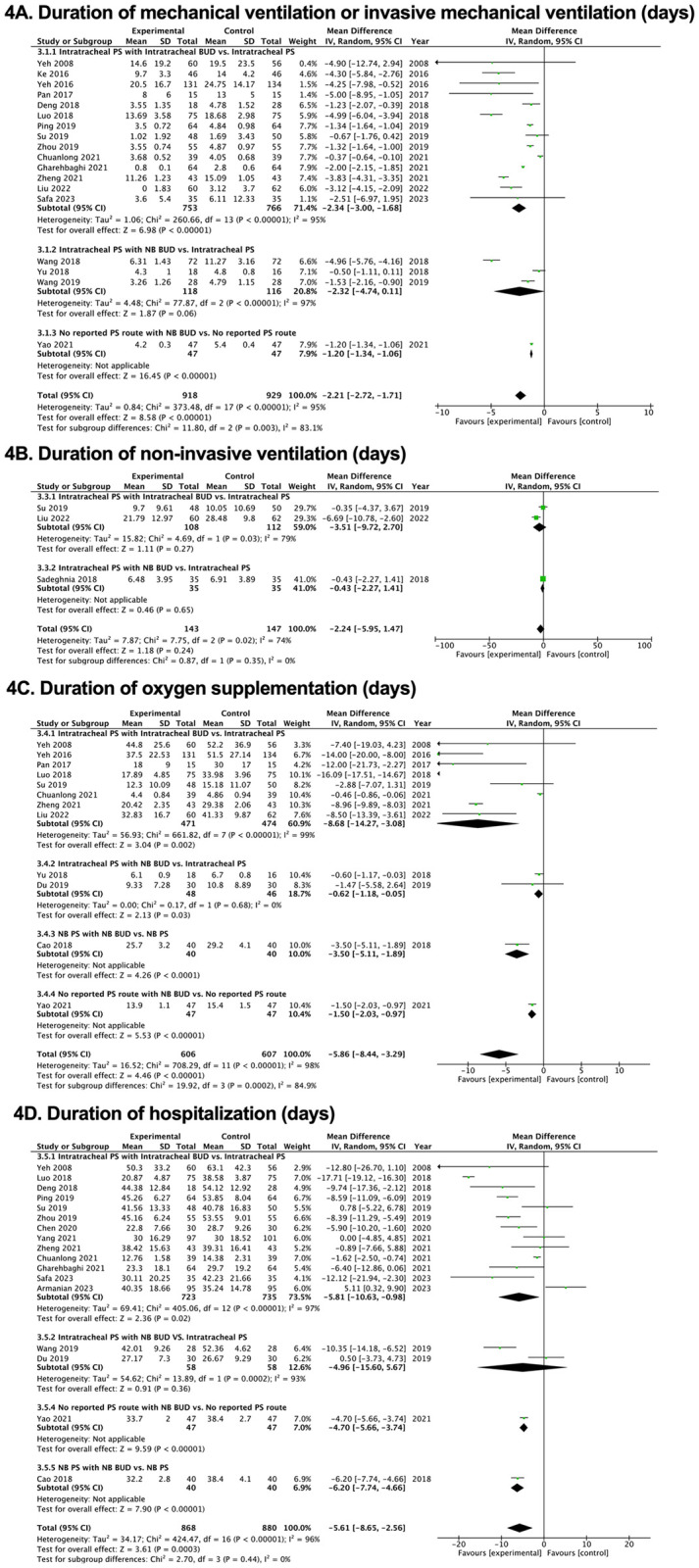
Forest plots of the effects of pulmonary surfactant with budesonide on clinical outcomes in neonates. (A) Duration of mechanical ventilation or invasive mechanical ventilation (days). (B) Duration of non-invasive ventilation (days). (C) Duration of oxygen supplementation (days). (D) Duration of hospitalization (days). BUD, budesonide; NB, nebulization; PS, pulmonary surfactant.

**Fig 5:**
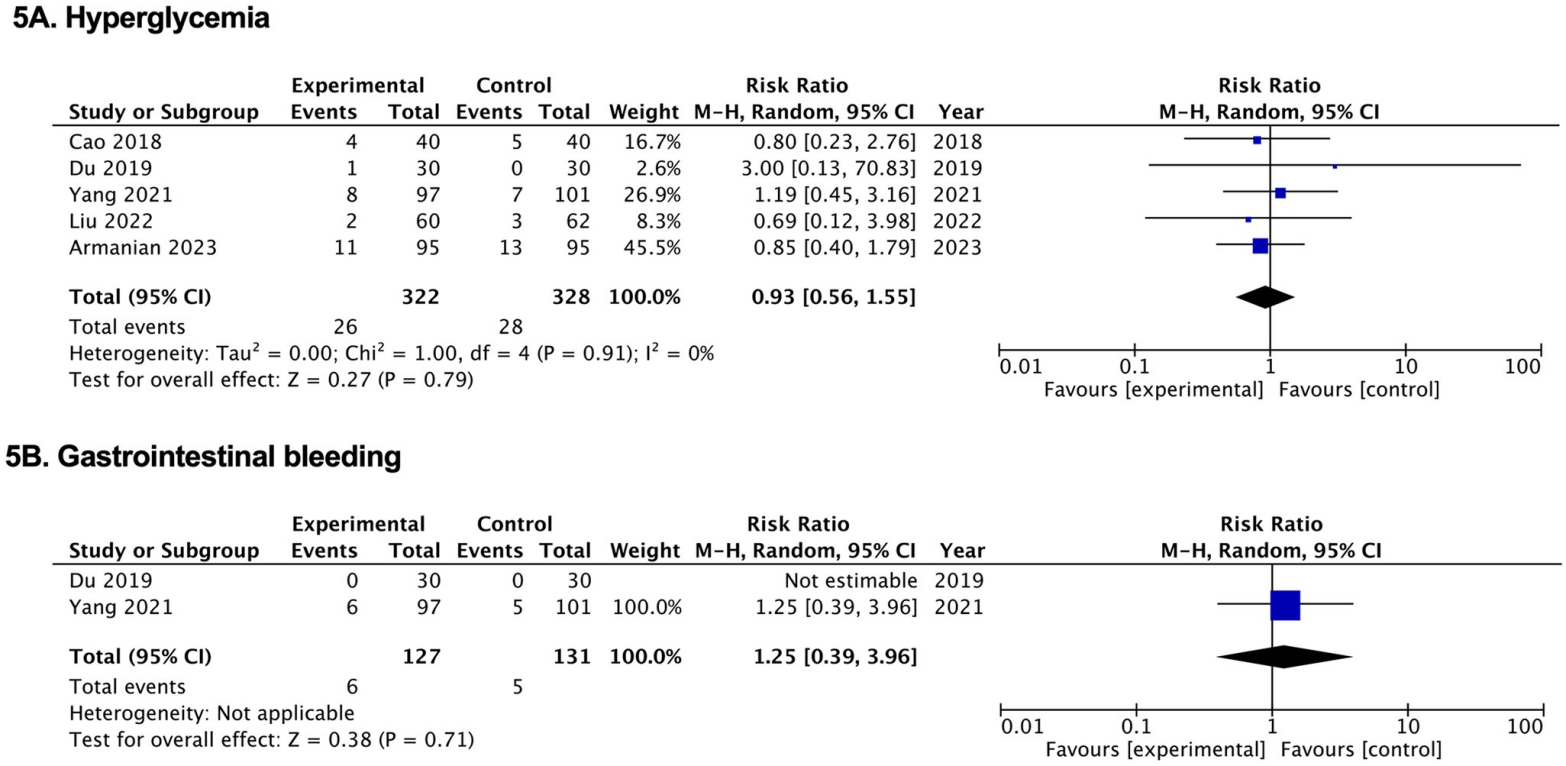
Forest plots of the effects of pulmonary surfactant with budesonide on adverse effects in neonates. (A) Hyperglycemia. (B) Gastrointestinal bleeding. BUD, budesonide; NB, nebulization; PS, pulmonary surfactant.

Figs 2–5, S2–S4 Tables and S2 and S3 Figs illustrate how PS and budesonide affect clinical outcomes and adverse effects in premature infants. Table 2 presents a GRADE summary of the findings on the efficacy of pulmonary surfactant with budesonide in premature infants.

**Table 2:** GRADE summary of the findings on the efficacy of pulmonary surfactant with budesonide in premature infants.

Patient or population: Preterm												
Intervention: Pulmonary surfactant with budesonide												
Comparison: Pulmonary surfactant												
Study design	No. of studies	Certainty assessment						No. of participants		Effect		
		Risk of bias	Inconsistency	Indirectness	Imprecision		Other considerations	PS with Budesonide	PS	Estimation of absolute effects		Certainty
										Risk (95% CI)	Absolute (95% CI)	
**Duration of mechanical ventilation or invasive mechanical ventilation (days)**												
RCT	18	serious ^a^	serious ^b^	not serious		not serious	none	918	929	-	MD **2.21 lower** (2.72 lower to 1.71 lower)	⨁⨁◯◯ Low
**Duration of non-invasive ventilation (days)**												
RCT	3	serious ^a^	serious ^b^	not serious		serious ^d^	none	143	147	-	MD **2.24 lower** (5.95 lower to 1.47 higher)	⨁◯◯◯ Very low
**Duration of oxygen supplementation (days)**												
RCT	12	serious ^a^	serious ^b^	not serious		not serious	publication bias strongly suspected	606	607	-	MD **5.86 lower** (8.44 lower to 3.29 lower)	⨁◯◯◯ Very low
**Duration of hospitalization (days)**												
RCT	17	serious ^a^	serious ^b^	not serious		not serious	none	868	880	-	MD **5.61 lower** (8.65 lower to 2.56 lower)	⨁⨁◯◯ Low
**Respiratory outcomes**												
**Incidence of bronchopulmonary dysplasia (BPD)**												
RCT	18	serious ^a^	not serious	not serious		not serious	publication bias strongly suspected	267/1097 (24.3%)	433/1104 (39.2%)	**RR 0.61** (0.51 to 0.73)	**153 fewer per 1,000** (from 192 fewer to 106 fewer)	⨁⨁◯◯ Low
**Incidence of moderate-to-severe BPD**												
RCT	8	serious ^a^	not serious	not serious		not serious	none	76/519 (14.6%)	170/541 (31.4%)	**RR 0.48** (0.36 to 0.63)	**163 fewer per 1,000** (from 201 fewer to 116 fewer)	⨁⨁⨁◯ Moderate
**Incidence of severe BPD**												
RCT	5	serious ^a^	not serious	not serious		not serious	none	15/372 (4.0%)	33/394 (8.4%)	**RR 0.49** (0.28 to 0.87)	**43 fewer per 1,000** (from 60 fewer to 11 fewer)	⨁⨁⨁◯ Moderate
**Redosing of pulmonary surfactant (PS)**												
RCT	12	serious ^a^	not serious	not serious		not serious	publication bias strongly suspected	122/686 (17.8%)	256/705 (36.3%)	**RR 0.50** (0.39 to 0.63)	**182 fewer per 1,000** (from 222 fewer to 134 fewer)	⨁⨁◯◯ Low
**Mortality**												
RCT	6	serious ^a^	not serious	not serious		serious ^d^	none	47/332 (14.2%)	64/328 (19.5%)	**RR 0.73** (0.48 to 1.09)	**53 fewer per 1,000** (from 101 fewer to 18 more)	⨁⨁◯◯ Low
**Switching to mechanical ventilation or invasive mechanical ventilation**												
RCT	6	serious ^a^	serious ^b^	not serious		serious ^d^	none	46/278 (16.5%)	76/278 (27.3%)	**RR 0.54** (0.27 to 1.07)	**126 fewer per 1,000** (from 200 fewer to 19 more)	⨁◯◯◯ Very low

^a^ Downgraded by one level for risk of bias due to unblinded outcome assessment

^b^ Downgraded by one level for inconsistency due to substantial heterogeneity (I^2^ = 50%).

^c^ Downgraded by one level due to indirect effects; potential influence from other factors

^d^ Downgraded by one level for imprecision as the 95% confidence interval includes both potential benefit and harm.

**Abbreviation:** CI: confidence interval; PS: Pulmonary surfactant; RCTs: Randomized controlled trials; RR: risk ratio

### Respiratory outcomes

#### Incidence of BPD

We analyzed the results from 18 RCTs [15–18, 30, 32–34, 36–38, 40, 43–48] involving 2,201 participants. Budesonide with PS reduced the incidence of BPD compared with that in the control group (RR, 0.61; 95% CI, 0.51, 0.73), with low-quality evidence certainty. Eight studies [15, 37–40, 43, 44, 47] found a significant difference in the incidence of moderate-to-severe BPD for budesonide with PS compared with the rate for the control group (RR, 0.48; 95% CI, 0.36, 0.63), with moderate evidence certainty. Five studies [15, 38, 43, 44, 47] demonstrated that budesonide with PS reduced the incidence of severe BPD compared with that in the control group (RR, 0.49; 95% CI, 0.28, 0.87), with moderate-quality evidence certainty (Table 2, Fig 2A–2C, S2 and S4 Tables and S3 Fig).

#### Redosing of PS

Twelve RCTs [15, 17, 28, 31, 33, 34, 36, 43, 44, 47–49] showed a statistically significant difference in the incidence of redosing PS in the intervention group compared with that in the control group (RR, 0.50; 95% CI, 0.39, 0.63), with low-quality evidence certainty (Table 2, Fig 2D, S2 and S4 Tables and S3 Fig).

#### Incidence of switching to mechanical ventilation (MV) or invasive mechanical ventilation (IMV) and MV or IMV duration

The findings regarding the need to switch to either MV or IMV as well as the duration of ventilators, were based on the terms used in each study. There was no statistically significant difference in the incidence of needing to switch to MV or IMV when budesonide was given with PS compared with that in the control group (RR, 0.54; 95% CI, 0.27, 1.07) in six RCTs [17, 28, 33, 34, 36, 43] with very low-quality evidence certainty. In 18 studies [15–17, 29, 31, 32, 34, 35, 37–40, 42, 43, 45–48], the length of MV or IMV significantly decreased when budesonide was given with PS (MD, −2.21 days; 95% CI, −2.72, −1.71), with low-quality evidence certainty (Table 2, Figs 3A and 4A, S2–S4 Tables and S3 Fig).

#### Reintubation

Compared with the rate in the control group, budesonide with PS did not significantly lower the number of reintubations in two RCTs [38, 48] (RR 0.87; 95% CI 0.50, 1.52) with low-quality evidence certainty (Fig 3C, S2 and S4 Tables).

#### Duration of non-invasive ventilation and oxygen supplementation

Three RCTs [33, 38, 47] showed that budesonide with PS did not significantly shorten the noninvasive ventilation time (MD, −2.24 days; 95% CI, −5.95, 1.47) with very low-quality evidence certainty. In 12 studies [15, 16, 29, 30, 32, 35, 36, 38, 42, 45–47], there was a substantial reduction in the duration of oxygen supplementation in the intervention group compared with that in the control group (MD, −5.86 days; 95% CI, −8.44, −3.29 days), with very low-quality evidence certainty (Table 2, Fig 4B and 4C, S2, S3 Tables and S3 Fig).

### Respiratory complications

#### Ventilator-associated pneumonia or respiratory infection, pneumothorax, and pulmonary hemorrhage

There was no statistically significant difference in the incidence of ventilator-associated pneumonia or respiratory infection, pneumothorax, and pulmonary hemorrhage when budesonide was administered with PS compared with that in the control group [(RR, 0.55; 95% CI, 0.27, 1.10), (RR, 1.13; 95% CI, 0.49, 2.57), and (RR, 0.64; 95% CI, 0.40, 1.02), respectively], with low to very low-quality evidence certainty (S2 and S4 Tables and S2(A)–S2(C) Fig).

### Neurological outcomes

#### Intraventricular hemorrhage, periventricular leukomalacia, and cerebral hemorrhage

There was no statistically significant difference in the incidence of intraventricular hemorrhage between budesonide with PS and the control group (RR, 1.06; 95% CI, 0.83, 1.35) in five RCTs [15, 18, 38, 48, 49], with very low-quality evidence certainty. The incidences of periventricular leukomalacia and cerebral hemorrhage were not significantly different between the two groups in two RCTs [38, 47] and four RCTs [30, 37, 40, 47] [(RR, 1.72; 95% CI, 0.42, 7.12) and (RR, 1.12; 95% CI, 0.89, 1.42), respectively], also with very low-quality evidence certainty (Fig 3D, S2 and S4 Tables and S2(D) and S2(E) Fig).

#### MDI ≤ 69 and PDI ≤ 69 scores and MDI and PDI scores at 2–3 years

Two RCTs [15, 27] comparing budesonide with PS to a control group found no significant difference in the incidences of MDI ≤ 69 (RR, 0.88; 95% CI, 0.57, 1.36) and PDI ≤ 69 (RR, 0.86; 95%, 0.58, 1.26), with low-quality evidence certainty. The MDI and PDI scores exhibited no significant differences in the intervention group compared with those in the control group [(MD, 2.38; 95% CI, −1.33, 6.10) and (MD, 1.83; 95%, −3.11, 6.76), respectively], with low-quality evidence certainty (S3 and S4 Tables and S2(F)–S2(I) Fig).

### Other preterm outcomes

The incidence of mortality was not significantly different between the group receiving budesonide with PS and the control group (RR, 0.73; 95% CI, 0.48, 1.09), with low-quality evidence certainty in six studies [16, 18, 30, 33, 48, 49]. Duration of hospitalization was significantly shortened in the budesonide with PS group (MD, −5.61 days; 95% CI, −8.65, −2.56), with low-quality evidence certainty from 17 RCTs [16, 18, 30–32, 36–46, 48] (Table 2, Figs 3B and 4D, S2–S4 Tables and S3 Fig).

There was no significant difference in the outcomes of retinopathy of prematurity, necrotizing enterocolitis, or sepsis when using PS with budesonide compared with the levels in the control, with very low-quality evidence certainty (S2 and S4 Tables, S2(J)–S2(L) and S3 Figs).

In 12 studies [15, 16, 18, 30, 33, 38, 43–48], the incidence of patent ductus arteriosus was significantly reduced in the PS with budesonide group (RR, 0.82; 95% CI, 0.72, 0.94), with very low-quality evidence certainty (S2 and S4 Tables, S2(M) and S3 Figs).

### Adverse effects

No significant differences in the incidence of hyperglycemia or gastrointestinal bleeding were observed between the groups [(RR, 0.93; 95% CI, 0.56, 1.55) and (RR, 1.25; 95% CI, 0.39, 3.96), respectively] (Fig 5A and 5B, S2 and S4 Tables).

## Discussion

Pulmonary surfactant lowers surface tension and optimizes lung function. As PS deficits worsen in premature newborns with RDS, there is an increase in the shear force of alveolus opening, which could lead to the development of BPD. Research has shown that steroids prevent BPD and enhance lung function. In this systematic review and meta-analysis, we compared the effects of PS with budesonide administered by any route to PS alone on BPD and respiratory outcomes, such as PS redosing and ventilation time. Other clinical outcomes included hospitalization time and preterm complications, such as intraventricular hemorrhage, retinopathy of prematurity, and necrotizing enterocolitis. Adverse effects, including hyperglycemia and gastrointestinal bleeding, were also monitored.

There has been long-standing discussion regarding the benefits of using posnatal steroids for respiratory outcomes and other complications associated with preterm birth. Early systemic dexamethasone and hydrocortisone have demonstrated beneficial effects in reducing death or BPD at 36 weeks [14, 50]. However, concerns were raised regarding an association between dexamethasone and cerebral palsy [51, 52]. The administration of steroids via inhalation or ITT instillation has been extensively investigated in the last few years. The use of PS as a vehicle for budesonide was also comprehensively studied by Yeh et al., who calculated the optimal ratio of PS to budesodine for use. There were studies that used budesonide in conjunction with PS (i.e., without mixing but administered at the same time) and may continue budesonide later on. In our study, we showed the benefits on BPD when PS and budesonide were coadministered compared with PS alone by either route (RR, 0.61; 95% CI, 0.51, 0.73). In a subgroup analysis of 13 trials, BPD decreased significantly when PS and budesonide were given intratracheally (RR, 0.64; 95% CI 0.52, 0.78), while in 3 studies, budesonide was given as an aerosol along with PS given intratracheally (RR, 0.55; 95% CI, 0.35, 0.86). One study on PS with budesonide as an aerosol (albeit without details on the route and dosage) revealed no significant decrease in the incidence of BPD compared with that in a control group (RR, 0.36; 95% CI, 0.12, 1.06). In one study comparing PS and budesonide as an aerosol with PS alone as an aerosol, the former was found to reduce the incidence of BPD (RR, 0.20; 95% CI, 0.05, 0.86). Overall, these studies show that, when PS was given with budesonide, the incidence of BPD decreased considerably compared with that when PS alone was administered. Notably, only the results on the incidence of moderate-to-severe BPD or severe BPD were supported by moderate-quality evidence certainty. Otherwise, the quality of evidence certainty was low to very low, including for other respiratory outcomes, such as redosing of PS, even the incidence of which was reduced for PS and budesonide compared with that for PS alone.

Administering PS and budesonide did not reduce the need to switch to MV or IMV. This means that the incidence of patients requiring escalation of respiratory support, such as a mechanical ventilator or invasive ventilation, was not decreased by this treatment combination, but the duration of MV or invasive MV decreased, as was the duration requiring oxygen. Although the duration of MV or IMV was shorter by 2.21 days in the group for which PS and budesonide were administered than in the group administered with PS alone, the main reduction occurred in the group for which PS and budesonide were administered intratracheally.

The overall need for non-invasive ventilation did not significantly decrease in the intervention group compared with the control group. Whether or not noninvasive treatment or oxygen therapy were used may depend on the practices at particular institutions. Variation in this regard could involve certain institutions continuing continuous positive airway pressure or oxygen supplement for a while, based on different criteria. However, the overall incidence of oxygen therapy decreased in the group for which combined therapy was administered, as did the overall hospitalization time by 5.61 days.

In a pooled estimate of studies, budesonide with PS did not reduce the reintubation rate or respiratory complications, such as pneumothorax and pulmonary hemorrhage, according to our meta-analysis. In addition, no increase in sepsis, ventilator-associated pneumonia, or respiratory infection was found compared with the rates in the control group. Therefore, administering budesonide may not improve, but it may also not increase the risk of these outcomes.

Tang et al. in 2021 [53] and Yi et al. in 2022 [54] conducted meta-analyses of 17 and 10 articles, respectively, to compare PS alone with PS and budesonide co-administration. In these studies and our own meta-analysis, PS with budesonide was found to significantly reduce the incidence of BPD, mechanical ventilation time, and hospital stay compared with PS alone but did not reduce the incidence of retinopathy of prematurity. The study by Tang et al. [53] and our study revealed significantly lower rates of PS redosing and patent ductus arteriosus when using PS with budesonide compared with the rates when using PS alone. However, this treatment combination did not reduce the incidence of intraventricular hemorrhage and did not significantly affect the MDI or PDI scores. Additionally, the results of our meta-analysis agreed with those of Yi et al. [54] regarding sepsis, necrotizing enterocolitis, and mortality, with these outcomes showing no significant reductions with the treatment combination.

As neurodevelopmental evaluation tools, the MDI examines adaptive behaviors, hand–eye coordination, visual and auditory perception, language abilities, cognitive ability, and exploratory activities [55, 56] whereas the PDI measures gross and fine motor movements [56]. In both of these tools, a score of ≤ 69 points is considered to reflect developmental delay [57]. The results of this study showed that PS plus budesonide had no impact on the MDI or PDI score, or on the incidence of cerebral hemorrhage or periventricular leukomalacia.

Our meta-analysis was conducted using the most recently published evidence. For inclusion in this systematic review and meta-analysis, the studies were mostly required to include subjects with a birth weight of less than 1500 g. There were 3 studies in which the subjects’ mean birth weight was less than 1000 g, 19 in which the mean birth weight was greater than 1000 g, and 4 studies in which there was no mean birth weight available but recruited the participants with the birth weight of 1500 g or less. The mean gestational age (GA) in the studies included here also correlated with birth weight; in the three studies with a lower mean birth weight, the mean GA was less than 28 weeks. The authors assumed that mean birth weight, or mean GA, may influence the effect of budesonide administration in conjunction with PS. These results suggest the need for further studies that consider the GA of the included subjects and their birth weight as variables to determine whether budesonide with PS exerts significant effects. Infants born extremely prematurely might benefit less from this therapeutic combination than those born at a later GA, as their lungs are more underdeveloped.

Currently, intensive research is ongoing worldwide; some of the studies are focusing on those born at less than 28 weeks, aimed at determining whether they will benefit from combining PS and budesonide. However, our study has demonstrated the benefits of administering budesonide in conjunction with PS to preterm populations, mostly in those with a GA of more than 28 weeks or a birth weight exceeding 1000 g, the evidence certainty for these findings is low to very low. Only results regarding the incidence of moderate-to-severe BPD or severe BPD were supported by moderate-quality evidence certainty.

### Strengths and limitations

We performed a meta-analysis of the outcomes of preterm infants following the administration of PS with budesonide or PS alone. This work involved a large pooled-estimate meta-analysis that included 26 RCTs (27 articles). However, this work has some limitations that should be mentioned here. For example, neonatal care, PS types, dosages, and administration routes may have varied across the analyzed trials. Second, most RCTs were conducted in China and thus involved ethnically similar subject groups. Thus, well-designed large-scale RCTs with consistent, homogeneous protocols should be performed to investigate the value of PS with budesonide in premature infants.

## Conclusions

The early administration of budesonide and PS may improve respiratory outcomes in premature infants. This combination significantly reduces the duration of mechanical ventilation or invasive mechanical ventilation, oxygen requirements, hospitalization, and incidences of BPD, patent ductus arteriosus, and PS redosing, with low to very low evidence certainty. Our findings suggest that these benefits come without significantly serious short-term side effects.

## Supporting information

S1 Table Baseline maternal and neonatal characteristics of the participants in the included studies. (DOCX)

S2 Table GRADE evidence profile of the evidence outcomes. (DOCX)

S3 Table Summary results of the included studies categorized by outcomes (continuous data). (DOCX)

S4 Table Summary results of the included studies categorized by outcomes (incidence). (DOCX)

S5 Table List of excluded studies and reasons for exclusion. (DOCX)

S1 Fig Risk of bias summary of the included studies using the revised Cochrane Risk of Bias Tool for Randomized Trials. (DOCX)

S2 Fig Results of the outcomes in the systematic review and meta-analysis. (DOCX)

S3 Fig Funnel plots. (DOCX)
