# Supplementary material for: Efficacy of pulmonary surfactant with budesonide in premature infants: A systematic review and meta-analysis
Source: PLoS One. 2025 Jan 9;20(1):e0312561. doi: 10.1371/journal.pone.0312561 (PMC11717239; doi:10.1371/journal.pone.0312561)
Supplement: S1 Table — (DOCX) [file pone.0312561.s001.docx]

**S1 Table. The baseline maternal and neonatal characteristics of the participants in the included studies**

| Author | Year | Number of participants | | GA (wks) | | Birth weight (g) | | Sex  Male/Female | |
| --- | --- | --- | --- | --- | --- | --- | --- | --- | --- |
|  |  | Intervention | Control | Intervention | Control | Intervention | Control | Intervention | Control |
| Yeh | 2008 | 60 | 56 | 26.40 ± 2.20 | 26.70 ± 2.30 | 881.00 ± 245.00 | 919.00 ± 272.00 | 31/29 | 29/27 |
| Kuo | 2010 | 35 | 32 | 26.90 ± 2.10 | 26.7 ± 2.5 | 947.00 ± 265.00 | 934.00 ± 243.00 | 20/15 | 16/16 |
| Wan | 2010 | 31 | 31 | 32.52 ± 2.69 | 31.98 ± 2.52 | 1580.50 ± 329.60 | 1601.30 ± 334.30 | 17/14 | 20/11 |
| Ke | 2016 | 46 | 46 | <32** | | <1500** | | NR** | |
| Yeh | 2016 | 131 | 134 | 26.50 ± 2.20 | 26.80 ± 2.20 | 882.00± 249.00 | 935.00 ± 283.00 | 71/60 | 72/62 |
| Pan | 2017 | 15 | 15 | 29.50 ± 1.800 | 30.00 ± 1.70 | 1260.00 ± 240.00 | 1360.00 ± 370.00 | 9/6 | 8/7 |
| Cao | 2018 | 40 | 40 | 30.10 ± 2.20 | 30.7 ± 1.80 | 1332.70 ± 109.30 | 1338.70 ± 105.30 | NR | NR |
| Deng | 2018 | 18 | 28 | NR** | | <1500** | | NR** | |
| Luo | 2018 | 75 | 75 | NR** | | 1160.75 ± 25.43 | 1169.87 ± 28.74 | 42/33 | 41/34 |
| Sadeghnia | 2018 | 35 | 35 | 26.90 ± 1.30 | 27.30 ± 0.80 | 902.90 ± 117.60 | 928.40 ± 143.70 | 17/17 | 15/20 |
| Wang | 2018 | 72 | 72 | 31.42 ± 4.27 | 31.51 ± 4.16 | 1934.54 ± 282.26 | 1972.54 ± 275.34 | 42/30 | 44/28 |
| Yu | 2018 | 18 | 16 | 31.40 ± 3.50** | | 1830.00 ± 720.00** | | 23/30** | |
| Du | 2019 | 30 | 30 | ≤ 32** | | ≤ 1500** | | NR** | |
| Ping | 2019 | 64 | 64 | 29.10 ± 1.24 | 28.93 ± 1.20 | 1264.93 ± 207.12 | 1260.33 ± 205.87 | 37/27 | 40/24 |
| Su | 2019 | 48 | 50 | 29.68 ± 1.55 | 29.16 ± 1.45 | 1351.35 ± 337.77 | 1211.80 ± 267.78 | 28/20 | 31/19 |
| Wang | 2019 | 28 | 28 | 29.51 ± 0.23 | 29.49 ± 0.27 | 1320.00 ± 150.00 | 1290.00 ± 210.00 | 12/16 | 14/14 |
| Zhou | 2019 | 55 | 55 | 29.37 ± 1.22 | 29.43 ± 1.25 | 1287.14 ± 209.25 | 1256.84 ± 204.81 | 32/23 | 34/21 |
| Chen | 2020 | 30 | 30 | ≤ 32** | | ≤ 1500** | | NR** | |
| Chuanlong | 2021 | 39 | 39 | 30.12 ± 3.06 | 31.81 ± 2.85 | 1710.00 ± 530.00 | 1680.00 ± 480.00 | 20/19 | 21/18 |
| Gharehbaghi | 2021 | 64 | 64 | 28.20 ± 1.70 | 28.40 ± 1.50 | 1055.00 ± 192.00 | 1089.00 ± 168.00 | 38/26 | 40/24 |
| Yang | 2021 | 97 | 101 | 30.60 ± 1.60 | 30.80 ± 1.70 | 1535.00 ± 351.00 | 1540.00 ± 340.00 | 49/48 | 50/51 |
| Yao | 2021 | 47 | 47 | 30.70 ± 1.70 | 30.50 ± 1.60 | 1325.63 ± 200.74 | 1350.48 ± 205.96 | 26/21 | 28/19 |
| Zheng | 2021 | 43 | 43 | 31.22 ± 1.04 | 30.43 ± 1.10 | 1658.69 ± 141.32 | 1662.27 ± 135.43 | 24/19 | 25/18 |
| Liu | 2022 | 60 | 62 | 29.60 ± 1.30 | 29.30 ± 1.40 | 1199.00 ± 137.00 | 1175.00 ±175.00 | 32/28 | 33/29 |
| Armanian | 2023 | 95 | 95 | 28.94 ± 1.57 | 29.02 ± 1.57 | 1134.97 ± 237.61 | 1190 ± 289.33 | 52/41 | 57/38 |
| Safa | 2023 | 35 | 35 | 29.94 ± 2.11 | 29.34 ± 2.19 | 1186.00 ± 224.14 | 1139.86 ± 230.28 | 16/19 | 18/17 |
| Marzban | 2024 | 67 | 67 | 31.66 ± 2.84 | 31.00 ± 2.96 | 1584.55 ± 505.02 | 1465.45 ± 520.88 | 29/38 | 40/27 |

**Abbreviations:** C: Control; CI: confidence interval; g: gram; GA: Gestational age; I: Intervention; NR: No report; SD: standard deviation

* Converted data from Median (IQR 1-3) to mean (SD)

** Average of both groups or the ratio of total

Data extractors: NP and MN; The date of data extraction: July 14-21, 2024
