# Supplementary material for: Efficacy of pulmonary surfactant with budesonide in premature infants: A systematic review and meta-analysis
Source: PLoS One. 2025 Jan 9;20(1):e0312561. doi: 10.1371/journal.pone.0312561 (PMC11717239; doi:10.1371/journal.pone.0312561)
Supplement: S2 Table — (DOCX) [file pone.0312561.s002.docx]

**S3 Table. GRADE evidence profile of the evidence outcomes**

| **Patient or population: Preterm**  **Intervention: Pulmonary surfactant with budesonide**  **Comparison: Pulmonary surfactant** | | | | | | | | | | | | | | | | |
| --- | --- | --- | --- | --- | --- | --- | --- | --- | --- | --- | --- | --- | --- | --- | --- | --- |
| **Study design** | **No. of studies** | **Certainty assessment** | | | | | | | | **No. of participants** | | | **Effect** | | | |
|  |  | **Risk of bias** | **Inconsistency** | | | **Indirectness** | **Imprecision** | | **Other considerations** | **PS with Budesonide** | **PS** | | **Estimation of absolute effects** | | | **Certainty** |
|  |  |  |  |  |  |  |  |  |  |  |  |  | **Risk**  **(95% CI)** | **Absolute (95% CI)** | |  |
| **Duration of mechanical ventilation or invasive mechanical ventilation (days)** | | | | | | | | | | | | | | | | |
| RCT | 18 | serious ^a^ | | serious ^b^ | not serious | | | not serious | none | 918 | 929 | - | | | MD **2.21 lower** (2.72 lower to 1.71 lower) | ⨁⨁◯◯ Low |
| **Duration of non-invasive ventilation (days)** | | | | | | | | | | | | | | | | |
| RCT | 3 | serious ^a^ | | serious ^b^ | not serious | | | serious ^d^ | none | 143 | 147 | - | | | MD **2.24 lower** (5.95 lower to 1.47 higher) | ⨁◯◯◯ Very low |
| **Duration of oxygen supplementation (days)** | | | | | | | | | | | | | | | | |
| RCT | 12 | serious ^a^ | | serious ^b^ | not serious | | | not serious | publication bias strongly suspected | 606 | 607 | - | | | MD **5.86 lower** (8.44 lower to 3.29 lower) | ⨁◯◯◯ Very low |
| **Duration of hospitalization (days)** | | | | | | | | | | | | | | | | |
| RCT | 17 | serious ^a^ | | serious ^b^ | not serious | | | not serious | none | 868 | 880 | - | | | MD **5.61 lower** (8.65 lower to 2.56 lower) | ⨁⨁◯◯ Low |
| **Mental Development Index (MDI) score** | | | | | | | | | | | | | | | | |
| RCT | 2 | not serious | | not serious | serious ^c^ | | | serious ^d^ | none | 120 | 119 | - | | | MD **2.38 higher** (1.33 lower to 6.1 higher) | ⨁⨁◯◯ Low |
| **Psychomotor Development Index (PDI) score** | | | | | | | | | | | | | | | | |
| RCT | 2 | not serious | | not serious | serious ^c^ | | | serious ^d^ | none | 120 | 119 | - | | | MD **1.83 higher** (3.11 lower to 6.76 higher) | ⨁⨁◯◯ Low |
| **Respiratory outcomes** | | | | | | | | | | | | | | | | |
| **Incidence of bronchopulmonary dysplasia (BPD)** | | | | | | | | | | | | | | | | |
| RCT | 18 | serious ^a^ | | not serious | not serious | | | not serious | publication bias strongly suspected | 267/1097 (24.3%) | 433/1104 (39.2%) | **RR 0.61** (0.51 to 0.73) | | | **153 fewer per 1,000** (from 192 fewer to 106 fewer) | ⨁⨁◯◯ Low |
| **Incidence of moderate-to-severe BPD** | | | | | | | | | | | | | | | | |
| RCT | 8 | serious ^a^ | | not serious | not serious | | | not serious | none | 76/519 (14.6%) | 170/541 (31.4%) | **RR 0.48** (0.36 to 0.63) | | | **163 fewer per 1,000** (from 201 fewer to 116 fewer) | ⨁⨁⨁◯ Moderate |
| **Incidence of severe BPD** | | | | | | | | | | | | | | | | |
| RCT | 5 | serious ^a^ | | not serious | not serious | | | not serious | none | 15/372 (4.0%) | 33/394 (8.4%) | **RR 0.49** (0.28 to 0.87) | | | **43 fewer per 1,000** (from 60 fewer to 11 fewer) | ⨁⨁⨁◯ Moderate |
| **Redosing of pulmonary surfactant (PS)** | | | | | | | | | | | | | | | | |
| RCT | 12 | serious ^a^ | | not serious | not serious | | | not serious | publication bias strongly suspected | 122/686 (17.8%) | 256/705 (36.3%) | **RR 0.50** (0.39 to 0.63) | | | **182 fewer per 1,000** (from 222 fewer to 134 fewer) | ⨁⨁◯◯ Low |
| **Mortality** | | | | | | | | | | | | | | | | |
| RCT | 6 | serious ^a^ | | not serious | not serious | | | serious ^d^ | none | 47/332 (14.2%) | 64/328 (19.5%) | **RR 0.73** (0.48 to 1.09) | | | **53 fewer per 1,000** (from 101 fewer to 18 more) | ⨁⨁◯◯ Low |
| **Switching to mechanical ventilation or invasive mechanical ventilation** | | | | | | | | | | | | | | | | |
| RCT | 6 | serious ^a^ | | serious ^b^ | not serious | | | serious ^d^ | none | 46/278 (16.5%) | 76/278 (27.3%) | **RR 0.54** (0.27 to 1.07) | | | **126 fewer per 1,000** (from 200 fewer to 19 more) | ⨁◯◯◯ Very low |
| **Reintubation** | | | | | | | | | | | | | | | | |
| RCT | 2 | serious ^a^ | | not serious | not serious | | | serious ^d^ | none | 16/83 (19.3%) | 19/85 (22.4%) | **RR 0.87** (0.50 to 1.52) | | | **29 fewer per 1,000** (from 112 fewer to 116 more) | ⨁⨁◯◯ Low |
| **Ventilator-associated pneumonia (VAP) or respiratory infection** | | | | | | | | | | | | | | | | |
| RCT | 5 | serious ^a^ | | not serious | serious ^c^ | | | serious ^d^ | none | 12/245 (4.9%) | 24/247 (9.7%) | **RR 0.55** (0.27 to 1.10) | | | **44 fewer per 1,000** (from 71 fewer to 10 more) | ⨁◯◯◯ Very low |
| **Pneumothorax** | | | | | | | | | | | | | | | | |
| RCT | 7 | serious ^a^ | | not serious | not serious | | | serious ^d^ | none | 24/441 (5.4%) | 17/447 (3.8%) | **RR 1.13** (0.49 to 2.57) | | | **5 more per 1,000** (from 19 fewer to 60 more) | ⨁⨁◯◯ Low |
| **Pulmonary hemorrhage** | | | | | | | | | | | | | | | | |
| RCT | 7 | serious ^a^ | | not serious | serious ^c^ | | | serious ^d^ | none | 30/438 (6.8%) | 49/444 (11.0%) | **RR 0.64** (0.40 to 1.02) | | | **40 fewer per 1,000** (from 66 fewer to 2 more) | ⨁◯◯◯ Very low |
| **Neurological outcomes** | | | | | | | | | | | | | | | | |
| **Intraventricular hemorrhage (IVH)** | | | | | | | | | | | | | | | | |
| RCT | 5 | serious ^a^ | | not serious | serious ^c^ | | | serious ^d^ | none | 140/376 (37.2%) | 135/381 (35.4%) | **RR 1.06** (0.83 to 1.35) | | | **21 more per 1,000** (from 60 fewer to 124 more) | ⨁◯◯◯ Very low |
| **Periventricular leukomalacia (PVL)** | | | | | | | | | | | | | | | | |
| RCT | 2 | serious ^a^ | | not serious | serious ^c^ | | | serious ^d^ | none | 5/108 (4.6%) | 3/112 (2.7%) | **RR 1.72** (0.42 to 7.12) | | | **19 more per 1,000** (from 16 fewer to 164 more) | ⨁◯◯◯ Very low |
| **Cerebral hemorrhage** | | | | | | | | | | | | | | | | |
| RCT | 4 | serious ^a^ | | not serious | serious ^c^ | | | serious ^d^ | none | 88/219 (40.2%) | 79/221 (35.7%) | **RR 1.12** (0.89 to 1.42) | | | **43 more per 1,000** (from 39 fewer to 150 more) | ⨁◯◯◯ Very low |
| **Mental Development Index (MDI) score** **≤ 69** | | | | | | | | | | | | | | | | |
| RCT | 2 | not serious | | not serious | serious ^c^ | | | serious ^d^ | none | 28/120 (23.3%) | 31/119 (26.1%) | **RR 0.88** (0.57 to 1.36) | | | **31 fewer per 1,000** (from 112 fewer to 94 more) | ⨁⨁◯◯ Low |
| **Psychomotor Development Index (PDI) score** **≤ 69** | | | | | | | | | | | | | | | | |
| RCT | 2 | not serious | | not serious | serious ^c^ | | | serious ^d^ | none | 34/120 (28.3%) | 39/119 (32.8%) | **RR 0.86** (0.58 to 1.26) | | | **46 fewer per 1,000** (from 138 fewer to 85 more) | ⨁⨁◯◯ Low |
| **Other preterm outcomes** | | | | | | | | | | | | | | | | |
| **Retinopathy of prematurity (ROP)** | | | | | | | | | | | | | | | | |
| RCT | 10 | serious ^a^ | | not serious | serious ^c^ | | | serious ^d^ | none | 158/616 (25.6%) | 175/620 (28.2%) | **RR 0.89** (0.75 to 1.05) | | | **31 fewer per 1,000** (from 71 fewer to 14 more) | ⨁◯◯◯ Very low |
| **Necrotizing enterocolitis (NEC)** | | | | | | | | | | | | | | | | |
| RCT | 9 | serious ^a^ | | not serious | serious ^c^ | | | serious ^d^ | none | 72/582 (12.4%) | 77/589 (13.1%) | **RR 0.95** (0.70 to 1.27) | | | **7 fewer per 1,000** (from 39 fewer to 35 more) | ⨁◯◯◯ Very low |
| **Sepsis** | | | | | | | | | | | | | | | | |
| RCT | 11 | serious ^a^ | | not serious | not serious | | | serious ^d^ | publication bias strongly suspected | 106/684 (15.5%) | 120/685 (17.5%) | **RR 0.89** (0.67 to 1.19) | | | **19 fewer per 1,000** (from 58 fewer to 33 more) | ⨁◯◯◯ Very low |
| **Patent Ductus Arteriosus (PDA)** | | | | | | | | | | | | | | | | |
| RCT | 12 | serious ^a^ | | not serious | serious ^c^ | | | not serious | publication bias strongly suspected | 217/698 (31.1%) | 274/701 (39.1%) | **RR 0.82** (0.72 to 0.94) | | | **70 fewer per 1,000** (from 109 fewer to 23 fewer) | ⨁◯◯◯ Very low |
| **Adverse effects** | | | | | | | | | | | | | | | | |
| **Hyperglycemia** | | | | | | | | | | | | | | | | |
| RCT | 5 | serious ^a^ | | not serious | not serious | | | serious ^d^ | none | 26/322 (8.1%) | 28/328 (8.5%) | **RR 0.93** (0.56 to 1.55) | | | **6 fewer per 1,000** (from 38 fewer to 47 more) | ⨁⨁◯◯ Low |
| **Gastrointestinal bleeding** | | | | | | | | | | | | | | | | |
| RCT | 2 | serious ^a^ | |  | serious ^c^ | | | not serious | none | 6/127 (4.7%) | 5/131 (3.8%) | **RR 1.25** (0.39 to 3.96) | | | **10 more per 1,000** (from 23 fewer to 113 more) | - |

^a^ Downgraded by one level for risk of bias due to unblinded outcome assessment
^b^ Downgraded by one level for inconsistency due to substantial heterogeneity (I² = 50%).

^c^ Downgraded by one level due to indirect effects; potential influence from other factors
^d^ Downgraded by one level for imprecision as the 95% confidence interval includes both potential benefit and harm.
**Abbreviation:** CI: confidence interval; PS: Pulmonary surfactant; RCTs: Randomized controlled trials; RR: risk ratio
