# Supplementary material for: Efficacy of pulmonary surfactant with budesonide in premature infants: A systematic review and meta-analysis
Source: PLoS One. 2025 Jan 9;20(1):e0312561. doi: 10.1371/journal.pone.0312561 (PMC11717239; doi:10.1371/journal.pone.0312561)
Supplement: S3 Table — (DOCX) [file pone.0312561.s003.docx]

**S4 Table. Summary results of the included studies categorized by outcomes (continuous data)**

| **Author (year)** | **Intervention** | **PS with budesonide** | | | | | | | **PS alone (control)** | | | | | | | **Mean difference (95% CI)** |
| --- | --- | --- | --- | --- | --- | --- | --- | --- | --- | --- | --- | --- | --- | --- | --- | --- |
|  |  | **Mean** | | | **SD** | | | **N** | **Mean** | | | | **SD** | | **N** |  |
| **Duration of mechanical ventilation or invasive mechanical ventilation (days)** | | | | | | | | | | | | | | | | |
| Yeh 2008 | PS ITT with budesonide ITT | | 14.60 | 19.20 | | | 60 | | | | 19.5 | 23.5 | | 56 | | -4.90 [-12.74, 2.94] |
| Ke 2016 | PS ITT with budesonide ITT | | 9.70 | 3.30 | | | 46 | | | | 14 | 4.20 | | 46 | | -4.30 [-5.84, -2.76] |
| Yeh 2016 | PS ITT with budesonide ITT | | 20.50* | 16.7* | | | 131 | | | | 24.75* | 14.17* | | 134 | | -4.25 [-7.98, -0.52] |
| Pan 2017 | PS ITT with budesonide ITT | | 8.00 | 6.00 | | | 15 | | | | 13.00 | 5.00 | | 15 | | -5.00 [-8.95, -1.05] |
| Deng 2018 | PS ITT with budesonide ITT | | 3.55 | 1.35 | | | 18 | | | | 4.78 | 1.52 | | 28 | | -1.23 [-2.07, -0.39] |
| Luo 2018 | PS ITT with budesonide ITT | | 13.69 | 3.58 | | | 75 | | | | 18.68 | 2.98 | | 75 | | -4.99 [-6.04, -3.94] |
| Wang 2018 | PS ITT with budesonide NB | | 6.31 | 1.43 | | | 72 | | | | 11.27 | 3.16 | | 72 | | -4.96 [-5.76, -4.16] |
| Yu 2018 | PS ITT with budesonide NB | | 4.30 | 1.00 | | | 18 | | | | 4.80 | 0.80 | | 16 | | -0.50 [-1.11, 0.11] |
| Ping 2019 | PS ITT with budesonide ITT | | 3.50 | 0.72 | | | 64 | | | | 4.84 | 0.98 | | 64 | | -1.34 [-1.64, -1.04] |
| Su 2019 | PS ITT with budesonide ITT | | 1.02 | 1.92 | | | 48 | | | | 1.69 | 3.43 | | 50 | | -0.67 [-1.76, 0.42] |
| Wang 2019 | PS ITT with budesonide NB | | 3.26 | 1.26 | | | 28 | | | | 4.79 | 1.15 | | 28 | | -1.53 [-2.16, -0.90] |
| Zhou 2019 | PS ITT with budesonide ITT | | 3.55 | 0.74 | | | 55 | | | | 4.87 | 0.97 | | 55 | | -1.32 [-1.64, -1.00] |
| Chuanlong 2021 | PS ITT with budesonide ITT | | 3.68 | 0.52 | | | 39 | | | | 4.05 | 0.68 | | 39 | | -0.37 [-0.64, -0.10] |
| Gharehbaghi 2021 | PS ITT with budesonide ITT | | 0.80 | 0.10 | | | 64 | | | | 2.80 | 0.60 | | 64 | | -2.00 [-2.15, -1.85] |
| Yao 2021 | PS no reported for dosage and route with budesonide NB | | 4.20 | 0.30 | | | 47 | | | | 5.40 | 0.40 | | 47 | | -1.20 [-1.34, -1.06] |
| Zheng 2021 | PS ITT with budesonide ITT | | 11.26 | 1.23 | | | 43 | | | | 15.09 | 1.05 | | 43 | | -3.83 [-4.31, -3.35] |
| Liu 2022 | PS ITT with budesonide ITT | | 0 | 1.83 | | | 60 | | | | 3.12 | 3.70 | | 62 | | -3.12 [-4.15, -2.09] |
| Safa 2023 | PS ITT with budesonide ITT | | 3.6 | 5.4 | | | 35 | | | | 6.11 | 12.33 | | 35 | | -2.51 [-6.97, 1.95] |
| Total (95% CI)  Heterogeneity: Tau² = 0.84; Chi² = 373.48, df = 17 (P < 0.00001); I² = 95%  Test for overall effect: Z = 8.58 (P < 0.00001) | | | | | | | | | | | | | | | | -2.21 [-2.72, -1.71] |
| **Duration of non-invasive ventilation (days)** | | | | | | | | | | | | | | | | |
| Sadeghnia 2018 | PS ITT with budesonide NB | 6.48 | | | 3.95 | 35 | | | | 6.91 | | | 3.89 | | 35 | -0.43 [-2.27, 1.41] |
| Su 2019 | PS ITT with budesonide ITT | 9.70 | | | 9.61 | 48 | | | | 10.05 | | | 10.69 | | 50 | -0.35 [-4.37, 3.67] |
| Liu 2022 | PS ITT with budesonide ITT | 21.79 | | | 12.97 | 60 | | | | 28.48 | | | 9.80 | | 62 | -6.69 [-10.78, -2.60] |
| Total (95% CI)  Heterogeneity: Tau² = 7.87; Chi² = 7.75, df = 2 (P = 0.02); I² = 74%  Test for overall effect: Z = 1.18 (P = 0.24) | | | | | | | | | | | | | | | | -2.24 [-5.95, 1.47] |
| **Duration of oxygen supplementation (days**) | | | | | | | | | | | | | | | | |
| Yeh 2008 | PS ITT with budesonide ITT | 44.80 | | | 25.60 | 60 | | | | 52.20 | | | 36.90 | | 56 | -7.40 [-19.03, 4.23] |
| Yeh 2016 | PS ITT with budesonide ITT | 37.50* | | | 22.53* | 131 | | | | 51.50* | | | 27.14* | | 134 | -14.00 [-20.00, -8.00] |
| Pan 2017 | PS ITT with budesonide ITT | 18.00 | | | 9.00 | 15 | | | | 30.00 | | | 17.00 | | 15 | -12.00 [-21.73, -2.27] |
| Cao 2018 | PS NB with budesonide NB | 25.70 | | | 3.20 | 40 | | | | 29.20 | | | 4.10 | | 40 | -3.50 [-5.11, -1.89] |
| Luo 2018 | PS ITT with budesonide ITT | 17.89 | | | 4.85 | 75 | | | | 33.98 | | | 3.96 | | 75 | -16.09 [-17.51, -14.67] |
| Yu 2018 | PS ITT with budesonide ITT | 6.10 | | | 0.90 | 18 | | | | 6.70 | | | 0.80 | | 16 | -0.60 [-1.17, -0.03] |
| Du 2019 | PS ITT with budesonide NB | 9.33 | | | 7.28 | 30 | | | | 10.8 | | | 8.89 | | 30 | -1.47 [-5.58, 2.64] |
| Su 2019 | PS ITT with budesonide ITT | 12.30 | | | 10.09 | 48 | | | | 15.18 | | | 11.07 | | 50 | -2.88 [-7.07, 1.31] |
| Chuanlong 2021 | PS ITT with budesonide ITT | 4.40 | | | 0.84 | 39 | | | | 4.86 | | | 0.94 | | 39 | -0.46 [-0.86, -0.06] |
| Yao 2021 | PS no reported for dosage and route with budesonide NB | 13.90 | | | 1.10 | 47 | | | | 15.40 | | | 1.50 | | 47 | -1.50 [-2.03, -0.97] |
| Zheng 2021 | PS ITT with budesonide ITT | 20.42 | | | 2.35 | 43 | | | | 29.38 | | | 2.06 | | 43 | -8.96 [-9.89, -8.03] |
| Liu 2022 | PS ITT with budesonide ITT | 32.83 | | | 16.7 | 60 | | | | 41.33 | | | 9.87 | | 62 | -8.50 [-13.39, -3.61] |
| Total (95% CI)  Heterogeneity: Tau² = 16.52; Chi² = 708.29, df = 11 (P < 0.00001); I² = 98%  Test for overall effect: Z = 4.46 (P < 0.00001) | | | | | | | | | | | | | | | | -5.86 [-8.44, -3.29] |
| **Duration of hospitalization (days)** | | | | | | | | | | | | | | | | |
| Yeh 2008 | PS ITT with budesonide ITT | 50.30 | | | 33.20 | 60 | | | | 63.10 | | | 42.30 | | 56 | -12.80 [-26.70, 1.10] |
| Cao 2018 | PS NB with budesonide NB | 32.20 | | | 2.80 | 40 | | | | 38.40 | | | 4.10 | | 40 | -6.20 [-7.74, -4.66] |
| Deng 2018 | PS ITT with budesonide ITT | 44.38 | | | 12.84 | 18 | | | | 54.12 | | | 12.92 | | 28 | -9.74 [-17.36, -2.12] |
| Luo 2018 | PS ITT with budesonide ITT | 20.87 | | | 4.87 | 75 | | | | 38.58 | | | 3.87 | | 75 | -17.71 [-19.12, -16.30] |
| Du 2019 | PS ITT with budesonide NB | 27.17 | | | 7.30 | 30 | | | | 26.67 | | | 9.29 | | 30 | 0.50 [-3.73, 4.73] |
| Ping 2019 | PS ITT with budesonide ITT | 45.26 | | | 6.27 | 64 | | | | 53.85 | | | 8.04 | | 64 | -8.59 [-11.09, -6.09] |
| Su 2019 | PS ITT with budesonide ITT | 41.56 | | | 13.33 | 48 | | | | 40.78 | | | 16.83 | | 50 | 0.78 [-5.22, 6.78] |
| Wang 2019 | PS ITT with budesonide NB | 42.01 | | | 9.26 | 28 | | | | 52.36 | | | 4.62 | | 28 | -10.35 [-14.18, -6.52] |
| Zhou 2019 | PS ITT with budesonide ITT | 45.16 | | | 6.24 | 55 | | | | 53.55 | | | 9.01 | | 55 | -8.39 [-11.29, -5.49] |
| Chen 2020 | PS ITT with budesonide ITT | 22.80 | | | 7.66 | 30 | | | | 28.70 | | | 9.26 | | 30 | -5.90 [-10.20, -1.60] |
| Chuanlong 2021 | PS ITT with budesonide ITT | 12.76 | | | 1.58 | 39 | | | | 14.38 | | | 2.31 | | 39 | -1.62 [-2.50, -0.74] |
| Gharehbaghi 2021 | PS ITT with budesonide ITT | 23.30 | | | 18.10 | 64 | | | | 29.70 | | | 19.20 | | 64 | -6.40 [-12.86, 0.06] |
| Yang 2021 | PS ITT with budesonide ITT | 30.00** | | | 16.29** | 97 | | | | 30.00** | | | 18.52** | | 101 | 0.00 [-4.85, 4.85] |
| Yao 2021 | PS no reported for dosage and route with budesonide NB | 33.70 | | | 2.00 | 47 | | | | 38.40 | | | 2.70 | | 47 | -4.70 [-5.66, -3.74] |
| Zheng 2021 | PS ITT with budesonide ITT | 38.42 | | | 15.63 | 43 | | | | 39.31 | | | 16.41 | | 43 | -0.89 [-7.66, 5.88] |
| Armanian 2023 | PS ITT with budesonide ITT | 40.35 | | | 18.66 | 95 | | | | 35.24 | | | 14.78 | | 95 | 5.11[0.32, 9.90] |
| Safa 2023 | PS ITT with budesonide ITT | 30.11 | | | 20.25 | 35 | | | | 42.23 | | | 21.66 | | 35 | -12.12 [-21.94, -2.30] |
| Total (95% CI)  Heterogeneity: Tau² = 34.17; Chi² = 424.47, df = 16 (P < 0.00001); I² = 96%  Test for overall effect: Z = 3.61 (P = 0.0003) | | | | | | | | | | | | | | | | -5.61 [-8.65, -2.56] |
| **Mental Development Index (MDI) score** | | | | | | | | | | | | | | | | |
| Kuo 2010 | PS ITT with budesonide ITT | 80.10 | | | 20.00 | 35 | | | | 74.90 | | | 20.60 | | 32 | 5.20 [-4.54, 14.94] |
| Yeh 2016 | PS ITT with budesonide ITT | 83.40 | | | 18.70 | 85 | | | | 81.50 | | | 2.80 | | 87 | 1.90 [-2.12, 5.92] |
| Total (95% CI)  Heterogeneity: Tau² = 0.00; Chi² = 0.38, df = 1 (P = 0.54); I² = 0%  Test for overall effect: Z = 1.26 (P = 0.21) | | | | | | | | | | | | | | | | 2.38 [-1.33, 6.10] |
| **Psychomotor Development Index (PDI) score** | | | | | | | | | | | | | | | | |
| Kuo 2010 | PS ITT with budesonide ITT | 79.90 | | | 20.80 | 35 | | | | 74.10 | | | 18.30 | | 32 | 5.80 [-3.56, 15.16] |
| Yeh 2016 | PS ITT with budesonide ITT | 77.90 | | | 18.70 | 85 | | | | 77.60 | | | 20.10 | | 87 | 0.30 [-5.50, 6.10] |
| Total (95% CI)  Heterogeneity: Tau² = 0.00; Chi² = 0.96, df = 1 (P = 0.33); I² = 0%  Test for overall effect: Z = 0.73 (P = 0.47) | | | | | | | | | | | | | | | | 1.83 [-3.11, 6.76] |

**Abbreviation:** CI: confidence interval; ITT: Intratracheal; NB: nebulization or inhalation; NR: No report; PS: Pulmonary surfactant; RCTs: Randomized controlled trials; RR: risk ratio

* Converted data from Median (range) to mean (SD)

** Converted data from Median (IQR 1-3) to mean (SD)
