# Supplementary material for: Efficacy of pulmonary surfactant with budesonide in premature infants: A systematic review and meta-analysis
Source: PLoS One. 2025 Jan 9;20(1):e0312561. doi: 10.1371/journal.pone.0312561 (PMC11717239; doi:10.1371/journal.pone.0312561)
Supplement: S4 Table — (DOCX) [file pone.0312561.s004.docx]

**S5 Table. Summary results of the included studies categorized by outcomes (incidence)**

| **Author (year)** | **Intervention** | **PS with budesonide** | | **Control ( PS alone)** | | **Risk Ratio  (95% CI)** |
| --- | --- | --- | --- | --- | --- | --- |
|  |  | **Event** | **Total** | **Event** | **Total** |  |
| **Respiratory outcomes** | | | | | | |
| Incidence of bronchopulmonary dysplasia (BPD) | | | | | | |
| Yeh 2008 | PS ITT with budesonide ITT | 9 | 60 | 16 | 56 | 0.53 [0.25, 1.09] |
| Yeh 2016 | PS ITT with budesonide ITT | 38 | 131 | 67 | 134 | 0.58 [0.42, 0.80] |
| Ke 2016 | PS ITT with budesonide ITT | 2 | 46 | 9 | 46 | 0.22 [0.05, 0.97] |
| Cao 2018 | PS NB with budesonide NB | 2 | 40 | 10 | 40 | 0.20 [0.05, 0.86] |
| Luo 2018 | PS ITT with budesonide ITT | 7 | 75 | 18 | 75 | 0.39 [0.17, 0.88] |
| Sadeghnia 2018 | PS ITT with budesonide NB | 11 | 35 | 19 | 35 | 0.58 [0.33, 1.03] |
| Wang 2018 | PS ITT with budesonide NB | 9 | 72 | 17 | 72 | 0.53 [0.25, 1.11] |
| Du 2019 | PS ITT with budesonide NB | 1 | 30 | 3 | 30 | 0.33 [0.04, 3.03] |
| Ping 2019 | PS ITT with budesonide ITT | 33 | 64 | 38 | 64 | 0.87 [0.64, 1.19] |
| Su 2019 | PS ITT with budesonide ITT | 20 | 48 | 33 | 50 | 0.63 [0.43, 0.93] |
| Zhou 2019 | PS ITT with budesonide ITT | 31 | 55 | 36 | 55 | 0.86 [0.64, 1.16] |
| Gharehbaghi 2021 | PS ITT with budesonide ITT | 20 | 64 | 38 | 64 | 0.53 [0.35, 0.80] |
| Yang 2021 | PS ITT with budesonide ITT | 11 | 97 | 14 | 101 | 0.82 [0.39, 1.71] |
| Yao 2021 | PS no reported for dosage and route with budesonide NB | 4 | 47 | 11 | 47 | 0.36 [0.12, 1.06] |
| Zheng 2021 | PS ITT with budesonide ITT | 3 | 43 | 13 | 43 | 0.23 [0.07, 0.75] |
| Liu 2022 | PS ITT with budesonide ITT | 17 | 60 | 29 | 62 | 0.61 [0.37, 0.98] |
| Armanian 2023 | PS ITT with budesonide ITT | 46 | 95 | 48 | 95 | 0.96 [0.72, 1.28] |
| Safa 2023 | PS ITT with budesonide ITT | 3 | 35 | 14 | 35 | 0.21 [0.07, 0.68] |
| Total (95% CI)  Heterogeneity: Tau² = 0.06; Chi² = 32.13, df = 17 (P = 0.01); I² = 47%  Test for overall effect: Z = 5.29 (P < 0.00001) | | | | | | 0.61 [0.51, 0.73] |
| **Incidence of moderate to severe BPD** | | | | | | |
| Yeh 2016 | PS ITT with budesonide ITT | 38 | 131 | 67 | 134 | 0.58 [0.42, 0.80] |
| Ping 2019 | PS ITT with budesonide ITT | 10 | 64 | 29 | 64 | 0.34 [0.18, 0.65] |
| Su 2019 | PS ITT with budesonide ITT | 6 | 20 | 13 | 33 | 0.76 [0.34, 1.68] |
| Wang 2019 | PS ITT with budesonide NB | 5 | 28 | 10 | 28 | 0.50 [0.20, 1.28] |
| Zhou 2019 | PS ITT with budesonide ITT | 9 | 55 | 30 | 55 | 0.30 [0.16, 0.57] |
| Gharehbaghi 2021 | PS ITT with budesonide ITT | 4 | 64 | 10 | 64 | 0.40 [0.13, 1.21] |
| Yang 2021 | PS ITT with budesonide ITT | 4 | 97 | 5 | 101 | 0.83 [0.23, 3.01] |
| Liu 2022 | PS ITT with budesonide ITT | 0 | 60 | 6 | 62 | 0.08 [0.00, 1.38] |
| Total (95% CI)  Heterogeneity: Tau² = 0.02; Chi² = 8.23, df = 7 (P = 0.31); I² = 15%  Test for overall effect: Z = 5.18 (P < 0.00001) | | | | | | 0.48 [0.36, 0.63] |
| **Incidence of severe BPD** | | | | | | |
| Yeh 2016 | PS ITT with budesonide ITT | 12 | 131 | 26 | 134 | 0.47 [0.25, 0.90] |
| Su 2019 | PS ITT with budesonide ITT | 1 | 20 | 3 | 33 | 0.55 [0.06, 4.93] |
| Gharehbaghi 2021 | PS ITT with budesonide ITT | 1 | 64 | 2 | 64 | 0.50 [0.05, 5.38] |
| Yang 2021 | PS ITT with budesonide ITT | 1 | 97 | 1 | 101 | 1.04 [0.07, 16.41] |
| Liu 2022 | PS ITT with budesonide ITT | 0 | 60 | 1 | 62 | 0.34 [0.01, 8.29] |
| Total (95% CI)  Heterogeneity: Tau² = 0.00; Chi² = 0.36, df = 4 (P = 0.99); I² = 0%  Test for overall effect: Z = 2.44 (P = 0.01) | | | | | | 0.49 [0.28, 0.87] |
| **Redosing of pulmonary surfactant (PS)** | | | | | | |
| Wan 2010 | PS ITT with budesonide ITT | 0 | 31 | 6 | 31 | 0.08 [0.00, 1.31] |
| Ke 2016 | PS ITT with budesonide ITT | 2 | 46 | 8 | 46 | 0.25 [0.06, 1.11] |
| Yeh 2016 | PS ITT with budesonide ITT | 46 | 131 | 85 | 134 | 0.55 [0.42, 0.72] |
| Deng 2018 | PS ITT with budesonide ITT | 0 | 18 | 2 | 28 | 0.31 [0.02, 6.01] |
| Sadeghnia 2018 | PS ITT with budesonide NB | 8 | 35 | 14 | 35 | 0.57 [0.27, 1.19] |
| Wang 2018 | PS ITT with budesonide NB | 6 | 72 | 19 | 72 | 0.32 [0.13, 0.74] |
| Du 2019 | PS ITT with budesonide NB | 2 | 30 | 4 | 30 | 0.50 [0.10, 2.53] |
| Gharehbaghi 2021 | PS ITT with budesonide ITT | 10 | 64 | 24 | 64 | 0.42 [0.22, 0.80] |
| Yang 2021 | PS ITT with budesonide ITT | 13 | 97 | 15 | 101 | 0.90 [0.45, 1.80] |
| Liu 2022 | PS ITT with budesonide ITT | 8 | 60 | 21 | 62 | 0.39 [0.19, 0.82] |
| Safa 2023 | PS ITT with budesonide ITT | 14 | 35 | 17 | 35 | 0.82 [0.48, 1.40] |
| Marzban 2024 | PS ITT with budesonide ITT | 13 | 67 | 41 | 37 | 0.32 [0.19, 0.54] |
| Total (95% CI)  Heterogeneity: Tau² = 0.04; Chi² = 14.53, df = 11 (P = 0.20); I² = 24%  Test for overall effect: Z = 5.76 (P < 0.00001) | | | | | | 0.50 [0.39, 0.63] |
| **Mortality** | | | | | | |
| Yeh 2008 | PS ITT with budesonide ITT | 10 | 60 | 18 | 56 | 0.52 [0.26, 1.03] |
| Cao 2018 | PS NB with budesonide NB | 1 | 40 | 2 | 40 | 0.50 [0.05, 5.30] |
| Sadeghnia 2018 | PS ITT with budesonide NB | 4 | 35 | 6 | 35 | 0.67 [0.21, 2.16] |
| Armanian 2023 | PS ITT with budesonide ITT | 13 | 95 | 15 | 95 | 0.87 [0.44, 1.72] |
| Safa 2023 | PS ITT with budesonide ITT | 9 | 35 | 4 | 35 | 2.25 [0.76, 6.63] |
| Marzban 2024 | PS ITT with budesonide ITT | 10 | 67 | 19 | 67 | 0.53 [0.26, 1.05] |
| Total (95% CI)  Heterogeneity: Tau² = 0.06; Chi² = 6.35, df = 5 (P = 0.27); I² = 21%  Test for overall effect: Z = 1.53 (P = 0.13) | | | | | | 0.73 [0.48, 1.09] |
| **Switching to mechanical ventilation or invasive mechanical ventilation** | | | | | | |
| Wan 2010 | PS ITT with budesonide ITT | 2 | 31 | 9 | 31 | 0.22 [0.05, 0.95] |
| Ke 2016 | PS ITT with budesonide ITT | 2 | 46 | 10 | 46 | 0.20 [0.05, 0.86] |
| Sadeghnia 2018 | PS ITT with budesonide NB | 9 | 35 | 4 | 35 | 2.25 [0.76, 6.63] |
| Wang 2018 | PS ITT with budesonide NB | 7 | 72 | 20 | 72 | 0.35 [0.16, 0.78] |
| Du 2019 | PS ITT with budesonide NB | 2 | 30 | 5 | 30 | 0.40 [0.08, 1.90] |
| Gharehbaghi 2021 | PS ITT with budesonide ITT | 24 | 64 | 28 | 64 | 0.86 [0.56, 1.31] |
| Total (95% CI)  Heterogeneity: Tau² = 0.43; Chi² = 14.42, df = 5 (P = 0.01); I² = 65%  Test for overall effect: Z = 1.76 (P = 0.08) | | | | | | 0.54 [0.27, 1.07] |
| **Reintubation** | | | | | |  |
| Su 2019 | PS ITT with budesonide ITT | 4 | 48 | 6 | 50 | 0.69 [0.21, 2.31] |
| Safa 2023 | PS ITT with budesonide ITT | 12 | 35 | 13 | 35 | 0.92 [0.49, 1.73] |
| Heterogeneity: Tau² = 0.00; Chi² = 0.17, df = 1 (P = 0.68); I² = 0%  Test for overall effect: Z = 0.50 (P = 0.62) | | | | | | 0.87 [0.50, 1.52] |
| **Ventilator-associated pneumonia (VAP) or respiratory infection** | | | | | | |
| Wan 2010 | PS ITT with budesonide ITT | 1 | 31 | 7 | 31 | 0.14 [0.02, 1.09] |
| Ping 2019 | PS ITT with budesonide ITT | 4 | 64 | 6 | 64 | 0.67 [0.20, 2.25] |
| Su 2019 | PS ITT with budesonide ITT | 1 | 48 | 1 | 50 | 1.04 [0.07, 16.19] |
| Zhou 2019 | PS ITT with budesonide ITT | 4 | 55 | 5 | 55 | 0.80 [0.23, 2.82] |
| Yao 2021 | PS no reported for dosage and route with budesonide NB | 2 | 47 | 5 | 47 | 0.40 [0.08, 1.96] |
| Total (95% CI)  Heterogeneity: Tau² = 0.00; Chi² = 2.55, df = 4 (P = 0.64); I² = 0%  Test for overall effect: Z = 1.69 (P = 0.09) | | | | | | 0.55 [0.27, 1.10] |
| **Pneumothorax** | | | | | | |
| Sadeghnia 2018 | PS ITT with budesonide NB | 2 | 35 | 1 | 35 | 2.00 [0.19, 21.06] |
| Su 2019 | PS ITT with budesonide ITT | 1 | 48 | 0 | 50 | 3.12 [0.13, 74.82] |
| Gharehbaghi 2021 | PS ITT with budesonide ITT | 1 | 64 | 3 | 64 | 0.33 [0.04, 3.12] |
| Yang 2021 | PS ITT with budesonide ITT | 1 | 97 | 1 | 101 | 1.04 [0.07, 16.41] |
| Armanian 2023 | PS ITT with budesonide ITT | 9 | 95 | 0 | 95 | 19.00 [1.12, 321.87] |
| Safa 2023 | PS ITT with budesonide ITT | 3 | 35 | 6 | 35 | 0.50 [0.14, 1.84] |
| Marzban 2024 | PS ITT with budesonide ITT | 7 | 67 | 6 | 67 | 1.17 [0.41, 3.29] |
| Total (95% CI)  Heterogeneity: Tau² = 0.27; Chi² = 7.71, df = 6 (P = 0.26); I² = 22%  Test for overall effect: Z = 0.28 (P = 0.78) | | | | | | 1.13 [0.49, 2.57] |
| **Pulmonary hemorrhage** | | | | | | |
| Ping 2019 | PS ITT with budesonide ITT | 8 | 64 | 6 | 64 | 1.33 [0.49, 3.63] |
| Su 2019 | PS ITT with budesonide ITT | 0 | 48 | 2 | 50 | 0.21 [0.01, 4.23] |
| Zhou 2019 | PS ITT with budesonide ITT | 6 | 55 | 5 | 55 | 1.20 [0.39, 3.70] |
| Gharehbaghi 2021 | PS ITT with budesonide ITT | 3 | 64 | 5 | 64 | 0.60 [0.15, 2.41] |
| Yang 2021 | PS ITT with budesonide ITT | 3 | 97 | 4 | 101 | 0.78 [0.18, 3.40] |
| Zheng 2021 | PS ITT with budesonide ITT | 2 | 43 | 6 | 43 | 0.33 [0.07, 1.56] |
| Marzban 2024 | PS ITT with budesonide ITT | 8 | 67 | 21 | 67 | 0.38 [0.18, 0.80] |
| Total (95% CI)  Heterogeneity: Tau² = 0.03; Chi² = 6.46, df = 6 (P = 0.37); I² = 7%  Test for overall effect: Z = 1.87 (P = 0.06) | | | | | | 0.64 [0.40, 1.02] |
| **Other preterm outcomes** | | | | | | |
| **Intraventricular hemorrhage (IVH)** | | | | | | |
| Yeh 2016 | PS ITT with budesonide ITT | 53 | 131 | 57 | 134 | 0.95 [0.71, 1.27] |
| Su 2019 | PS ITT with budesonide ITT | 3 | 48 | 1 | 50 | 3.13 [0.34, 29.01] |
| Armanian 2023 | PS ITT with budesonide ITT | 56 | 95 | 45 | 95 | 1.24 [0.95, 1.63] |
| Safa 2023 | PS ITT with budesonide ITT | 19 | 35 | 16 | 35 | 1.19 [0.74, 1.90] |
| Marzban 2024 | PS ITT with budesonide ITT | 9 | 67 | 16 | 67 | 0.56 [0.27, 1.18] |
| Total (95% CI)  Heterogeneity: Tau² = 0.02; Chi² = 5.88, df = 4 (P = 0.21); I² = 32%  Test for overall effect: Z = 0.45 (P = 0.66) | | | | | | 1.06 [0.83, 1.35] |
| **Periventricular leukomalacia (PVL)** | | | | | | |
| Su 2019 | PS ITT with budesonide ITT | 1 | 48 | 1 | 50 | 1.04 [0.07, 16.19] |
| Liu 2022 | PS ITT with budesonide ITT | 4 | 60 | 2 | 62 | 2.07 [0.39, 10.87] |
| Total (95% CI)  Heterogeneity: Tau² = 0.00; Chi² = 0.18, df = 1 (P = 0.68); I² = 0%  Test for overall effect: Z = 0.75 (P = 0.45) | | | | | | 1.72 [0.42, 7.12] |
| **Cerebral hemorrhage** | | | | | | |
| Cao 2018 | PS NB with budesonide NB | 14 | 40 | 11 | 40 | 1.27 [0.66, 2.45] |
| Ping 2019 | PS ITT with budesonide ITT | 28 | 64 | 25 | 64 | 1.12 [0.74, 1.69] |
| Zhou 2019 | PS ITT with budesonide ITT | 26 | 55 | 23 | 55 | 1.13 [0.74, 1.72] |
| Liu 2022 | PS ITT with budesonide ITT | 20 | 60 | 20 | 62 | 1.03 [0.62, 1.72] |
| Total (95% CI)  Heterogeneity: Tau² = 0.00; Chi² = 0.24, df = 3 (P = 0.97); I² = 0%  Test for overall effect: Z = 0.95 (P = 0.34) | | | | | | 1.12 [0.89, 1.42] |
| **Mental Development Index (MDI) score ≤ 69** | | | | | | |
| Kuo 2010 | PS ITT with budesonide ITT | 10 | 35 | 12 | 32 | 0.76 [0.38, 1.52] |
| Yeh 2016 | PS ITT with budesonide ITT | 18 | 85 | 19 | 87 | 0.97 [0.55, 1.72] |
| Total (95% CI)  Heterogeneity: Tau² = 0.00; Chi² = 0.28, df = 1 (P = 0.60); I² = 0%  Test for overall effect: Z = 0.58 (P = 0.57) | | | | | | 0.88 [0.57, 1.36] |
| **Psychomotor Development Index (PDI) score ≤ 69** | | | | | | |
| Kuo 201 | PS ITT with budesonide ITT | 10 | 35 | 13 | 32 | 0.70 [0.36, 1.38] |
| Yeh 2016 | PS ITT with budesonide ITT | 24 | 85 | 26 | 87 | 0.94 [0.59, 1.51] |
| Total (95% CI)  Heterogeneity: Tau² = 0.00; Chi² = 0.50, df = 1 (P = 0.48); I² = 0%  Test for overall effect: Z = 0.78 (P = 0.43) | | | | | | 0.86 [0.58, 1.26] |
| **Retinopathy of prematurity (ROP)** | | | | | | |
| Yeh 2008 | PS ITT with budesonide ITT | 25 | 60 | 21 | 56 | 1.11 [0.71, 1.75] |
| Cao 2018 | PS NB with budesonide NB | 4 | 40 | 4 | 40 | 1.00 [0.27, 3.72] |
| Ping 2019 | PS ITT with budesonide ITT | 22 | 64 | 25 | 64 | 0.88 [0.56, 1.39] |
| Su 2019 | PS ITT with budesonide ITT | 6 | 48 | 2 | 50 | 3.13 [0.66, 14.73] |
| Zhou 2019 | PS ITT with budesonide ITT | 21 | 55 | 25 | 55 | 0.84 [0.54, 1.31] |
| Gharehbaghi 2021 | PS ITT with budesonide ITT | 2 | 64 | 3 | 64 | 0.67 [0.12, 3.86] |
| Yang 2021 | PS ITT with budesonide ITT | 23 | 95 | 25 | 99 | 0.96 [0.59, 1.57] |
| Liu 2022 | PS ITT with budesonide ITT | 6 | 60 | 7 | 62 | 0.89 [0.32, 2.48] |
| Armanian 2023 | PS ITT with budesonide ITT | 37 | 95 | 41 | 95 | 0.90 [0.64, 1.27] |
| Safa 2023 | PS ITT with budesonide ITT | 12 | 35 | 22 | 35 | 0.55 [0.32, 0.92] |
| Total (95% CI)  Heterogeneity: Tau² = 0.00; Chi² = 7.12, df = 9 (P = 0.62); I² = 0%  Test for overall effect: Z = 1.36 (P = 0.17) | | | | | | 0.89 [0.75, 1.05] |
| **Necrotizing enterocolitis (NEC)** | | | | | | |
| Yeh 2016 | PS ITT with budesonide ITT | 4 | 131 | 7 | 134 | 0.58 [0.18, 1.95] |
| Du 2019 | PS ITT with budesonide NB | 2 | 30 | 1 | 30 | 2.00 [0.19, 20.90] |
| Ping 2019 | PS ITT with budesonide ITT | 13 | 64 | 11 | 64 | 1.18 [0.57, 2.44] |
| Su 2019 | PS ITT with budesonide ITT | 6 | 48 | 3 | 50 | 2.08 [0.55, 7.86] |
| Zhou 2019 | PS ITT with budesonide ITT | 12 | 55 | 10 | 55 | 1.20 [0.57, 2.54] |
| Gharehbaghi 2021 | PS ITT with budesonide ITT | 2 | 64 | 4 | 64 | 0.50 [0.09, 2.63] |
| Liu 2022 | PS ITT with budesonide ITT | 7 | 60 | 9 | 62 | 0.80 [0.32, 2.02] |
| Armanian 2023 | PS ITT with budesonide ITT | 20 | 95 | 20 | 95 | 1.00 [0.58, 1.73] |
| Safa 2023 | PS ITT with budesonide ITT | 6 | 35 | 12 | 35 | 0.50 [0.21, 1.18] |
| Total (95% CI)  Heterogeneity: Tau² = 0.00; Chi² = 5.94, df = 8 (P = 0.65); I² = 0%  Test for overall effect: Z = 0.37 (P = 0.71) | | | | | | 0.95 [0.70, 1.27] |
| **Sepsis** | | | | | | |
| Yeh 2008 | PS ITT with budesonide ITT | 6 | 60 | 5 | 56 | 1.12 [0.36, 3.47] |
| Yeh 2016 | PS ITT with budesonide ITT | 29 | 131 | 38 | 134 | 0.78 [0.51, 1.19] |
| Du 2019 | PS ITT with budesonide NB | 2 | 30 | 2 | 30 | 1.00 [0.15, 6.64] |
| Ping 2019 | PS ITT with budesonide ITT | 0 | 64 | 2 | 64 | 0.20 [0.01, 4.09] |
| Zhou 2019 | PS ITT with budesonide ITT | 0 | 55 | 2 | 55 | 0.20 [0.01, 4.07] |
| Gharehbaghi 2021 | PS ITT with budesonide ITT | 21 | 64 | 25 | 64 | 0.84 [0.53, 1.34] |
| Yao 2021 | PS no reported for dosage and route with budesonide NB | 0 | 47 | 2 | 47 | 0.20 [0.01, 4.06] |
| Zheng 2021 | PS ITT with budesonide ITT | 2 | 43 | 5 | 43 | 0.40 [0.08, 1.95] |
| Liu 2022 | PS ITT with budesonide ITT | 19 | 60 | 22 | 62 | 0.89 [0.54, 1.47] |
| Armanian 2023 | PS ITT with budesonide ITT | 22 | 95 | 9 | 95 | 2.44 [1.19, 5.03] |
| Safa 2023 | PS ITT with budesonide ITT | 5 | 35 | 8 | 35 | 0.63 [0.23, 1.72] |
| Total (95% CI)  Heterogeneity: Tau² = 0.04; Chi² = 12.42, df = 10 (P = 0.26); I² = 19%  Test for overall effect: Z = 0.76 (P = 0.45) | | | | | | 0.89 [0.67, 1.19] |
| **Patent Ductus Arteriosus (PDA)** | | | | | | |
| Yeh 2008 | PS ITT with budesonide ITT | 36 | 60 | 32 | 56 | 1.05 [0.77, 1.43] |
| Yeh 2016 | PS ITT with budesonide ITT | 40 | 131 | 59 | 134 | 0.69 [0.50, 0.96] |
| Cao 2018 | PS NB with budesonide NB | 3 | 40 | 3 | 40 | 1.00 [0.21, 4.66] |
| Sadeghnia 201 | PS ITT with budesonide NB | 8 | 35 | 11 | 35 | 0.73 [0.33, 1.59] |
| Su 2019 | PS ITT with budesonide ITT | 7 | 48 | 19 | 50 | 0.38 [0.18, 0.83] |
| Gharehbaghi 2021 | PS ITT with budesonide ITT | 13 | 64 | 17 | 64 | 0.76 [0.41, 1.44] |
| Yang 2021 | PS ITT with budesonide ITT | 21 | 40 | 23 | 40 | 0.91 [0.61, 1.36] |
| Yao 2021 | PS no reported for dosage and route with budesonide NB | 0 | 47 | 1 | 47 | 0.33 [0.01, 7.98] |
| Zheng 2021 | PS ITT with budesonide ITT | 3 | 43 | 5 | 43 | 0.60 [0.15, 2.36] |
| Liu 2022 | PS ITT with budesonide ITT | 29 | 60 | 31 | 62 | 0.97 [0.67, 1.39] |
| Armanian 2023 | PS ITT with budesonide ITT | 38 | 95 | 47 | 95 | 0.81 [0.59, 1.11] |
| Safa 2023 | PS ITT with budesonide ITT | 19 | 35 | 26 | 35 | 0.73 [0.51, 1.05] |
| Total (95% CI)  Heterogeneity: Tau² = 0.00; Chi² = 9.73, df = 11 (P = 0.55); I² = 0%  Test for overall effect: Z = 2.95 (P = 0.003) | | | | | | 0.82 [0.72, 0.94] |
| **Adverse effects** | | | | | |  |
| **Hyperglycemia** | | | | | | |
| Cao 2018 | PS NB with budesonide NB | 4 | 40 | 5 | 40 | 0.80 [0.23, 2.76] |
| Du 2019 | PS ITT with budesonide NB | 1 | 30 | 0 | 30 | 3.00 [0.13, 70.83] |
| Yang 2021 | PS ITT with budesonide ITT | 8 | 97 | 7 | 101 | 1.19 [0.45, 3.16] |
| Liu 2022 | PS ITT with budesonide ITT | 2 | 60 | 3 | 62 | 0.69 [0.12, 3.98] |
| Armanian 2023 | PS ITT with budesonide ITT | 11 | 95 | 13 | 95 | 0.85 [0.40, 1.79] |
| Total (95% CI)  Heterogeneity: Tau² = 0.00; Chi² = 1.00, df = 4 (P = 0.91); I² = 0%  Test for overall effect: Z = 0.27 (P = 0.79) | | | | | | 0.93 [0.56, 1.55] |
| **Gastrointestinal bleeding** | | | | | | |
| Du 2019 | PS ITT with budesonide NB | 0 | 30 | 0 | 30 | Not estimable |
| Yang 2021 | PS ITT with budesonide ITT | 6 | 97 | 5 | 101 | 1.25 [0.39, 3.96] |
| Total (95% CI)  Heterogeneity: Not applicable  Test for overall effect: Z = 0.38 (P = 0.71) | | | | | | 1.25 [0.39, 3.96] |

**Abbreviation:** CI: confidence interval; ITT: Intratracheal; NB: nebulization or inhalation; PS: Pulmonary surfactant; RCTs: Randomized controlled trials; RR: risk ratio
