# Supplementary material for: Efficacy of pulmonary surfactant with budesonide in premature infants: A systematic review and meta-analysis
Source: PLoS One. 2025 Jan 9;20(1):e0312561. doi: 10.1371/journal.pone.0312561 (PMC11717239; doi:10.1371/journal.pone.0312561)
Supplement: S5 Table — (DOCX) [file pone.0312561.s005.docx]

**S8 Table. List of excluded studies and reasons for exclusion**

| **Author** | **Year** | **Study name** | **DOI** | **Journal** | **URL** | **Reasons for exclusion** |
| --- | --- | --- | --- | --- | --- | --- |
| Arnon | 1996 | Effectiveness of budesonide aerosol in ventilator-dependent preterm babies: A preliminary report | 10.1002/(sici)1099-0496(199604)21:4<231::Aid-ppul5>3.0.Co;2-r. | Pediatric Pulmonology - Volume 21, Issue 4, pp. 231-235 - published 1996-01-01. | [https://www.embase.com/search/results?subaction=viewrecord&id=L26134830&from=exporthttp://dx.doi.org/10.1002/(SICI)1099-0496(199604)21:4<231::AID-PPUL5>3.0.CO;2-R](https://www.embase.com/search/results?subaction=viewrecord&id=L26134830&from=exporthttp://dx.doi.org/10.1002/(SICI)1099-0496(199604)21:4%3c231::AID-PPUL5%3e3.0.CO;2-R) | Irrevant  No surfactant use in the study |
| Inwald | 1999 | The effect of early inhaled budesonide on pulmonary inflammation in infants with respiratory distress syndrome | doi:10.1007/s004310051212 | European Journal of Pediatrics | https://www.scopus.com/inward/record.uri?eid=2-s2.0-0032836310&doi=10.1007%2fs004310051212&partnerID=40&md5=f97c9bba6661445f4a1d078d9fd68044 | Not RCT  This study was not a control trial |
| Bassler | 2010 | The Neonatal European Study of Inhaled Steroids (NEUROSIS): an eu-funded international randomised controlled trial in preterm infants | DOI: 10.1159/000227294 | *Neonatology* (2009) 97 (1): 52–55. | <https://karger.com/neo/article-abstract/97/1/52/228347/The-Neonatal-European-Study-of-Inhaled-Steroids?redirectedFrom=fulltext> | Protocol  This was a protocol for study in comparison of budesonide with placebo |
| Wu, S.-Y. | 2012 | Budesonide therapy in preterm infants to prevent bronchopulmonary dysplasia | 10.1542/neo.13-8-e467. | NeoReviews - Volume 13, Issue 8, pp. e467-e475 - published 2012-01-01. | <https://www.scopus.com/inward/record.uri?eid=2-s2.0-84865659819&doi=10.1542%2fneo.13-8-e467&partnerID=40&md5=abc9ab561e0be0ae2c242b162e600b04> | Not RCT  This is a review |
| McEvoy, C.T | 2019 | Pilot dose escalation trial of budesonide in surfactant in ventilated extremely low gestational age newborns | doi:10.1136/jim-2018-000939.266 | Journal of Investigative Medicine | <https://www.embase.com/search/results?subaction=viewrecord&id=L626915733&from=exporthttp://dx.doi.org/10.1136/jim-2018-000939.266> | Irrelevant study  This was comparing in the different doses of budesonide in surfactant for prevention of bronchopulmonary dysplasia in extremely low gestational age high-risk newborns (SASSIE). |
| Heo | 2020 | Intratracheal administration of budesonide with surfactant in very low birth weight infants to prevent bronchopulmonary dysplasia | NeoReviews - Volume 13, Issue 8, pp. e467-e475 - published 2012-01-01. | The Turkish Journal of Pediatrics 2020; 62: 551-559 | <https://pubmed.ncbi.nlm.nih.gov/32779407/> | This was no RCT study. |
| McEvoy, C.T | 2020 | Dose-escalation trial of budesonide in surfactant for prevention of bronchopulmonary dysplasia in extremely low gestational age high-risk newborns (SASSIE) | doi:10.1038/s41390-020-0792-y | Pediatr Res | <https://www.nature.com/articles/s41390-020-0792-y> | Irrelevant study  This study was a comparison of the deifferent doses of budesonide study and there was no control as the no budesonide group. |
| Tukova | 2020 | Early inhaled budesonide in extremely preterm infants decreases long-term respiratory morbidity | doi:10.1002/ppul.24704 | Pediatric Pulmonology | <https://www.scopus.com/inward/record.uri?eid=2-s2.0-85081016882&doi=10.1002%2fppul.24704&partnerID=40&md5=7fa9dd69b622350b7d7e780a29558cec> | Irrelevant study  This study compared budesonide with placebo, not budesonide with PS |
| Ballard P | 2021 | Blood metabolomics in infants enrolled in a dose escalation pilot trial of budesonide in surfactant | doi: 10.1038/s41390-020-01343-z. | Pediatr Res | <https://pubmed.ncbi.nlm.nih.gov/33469180/> | Irrelevant study  This study was a pilot dose escalation trial of budesonide in surfactant to intubated premature infants. |
| Gurung | 2023 | Neurodevelopmental outcomes in extremely premature infants treated with intratracheal budesonide-surfactant for the prevention of bronchopulmonary dysplasia | doi:10.1016/S0002-9629(23)00609-2 | American journal of the medical sciences | <https://www.cochranelibrary.com/central/doi/10.1002/central/CN-02522137/full> | Not RCT  This was a retrospective study. |
| Kashaki 2023 | 2023 | Simultaneous intratracheal administration of surfactant and  budesonide in the prevention of bronchopulmonary dysplasia  in the treatment of respiratory distress syndrome in  premature infants |  | Advancements in Life Sciences \| www.als-journal.com \| June 2023 \| Volume 10 \| Issue 2 | <https://www.als-journal.com/articles/vol10issue2/10225.23/1856.pdf> | Invalid data  The data in this study was invalid. |
| Manley | 2023 | Intratracheal budesonide mixed with surfactant to increase survival free of bronchopulmonary dysplasia in extremely preterm infants: study protocol for the international, multicenter, randomized PLUSS trial | doi:10.1186/s13063-023-07257-5 | Trials, 2023, 24(1), 320 \| added to CENTRAL: 31 May 2023 \| 2023 Issue 5 | https://www.cochranelibrary.com/central/doi/10.1002/central/CN-02562155/full | Protocol  This was a protocol for study in comparison of budesonide with surfactant and surfactant alone. |
| Habibi | 2024 | Comparison of the Consequences of Intratracheal Administration of  Surfactant Plus Budesonide with Surfactant alone in Preterm Infants with  Respiratory Distress Syndrome | ‎ 10.61186/sjku.28.6.48 | **Volume 28, Issue 6 (Scientific Journal of Kurdistan University of Medical Sciences 2024)**    SJKU 2024; 28 (6) :48-64 | <http://sjku.muk.ac.ir/article-1-7342-en.html> | Invalid data  The data in this study was invalid. |
| Nobile | 2024 | Long-term Effects of Intratracheal Budesonide and Surfactant for the Prevention of Bronchopulmonary Dysplasia: A Narrative Review. | 10.1055/s-0043-1769795. | American journal of perinatology - Volume 41, Issue 0, pp. e1858-e1865 - published 2024-05-01. | <https://pubmed.ncbi.nlm.nih.gov/37279790/> | This was a systematic review study. |
| Prodanovic | 2024 | Advanced Diagnostics of Respiratory Distress Syndrome in Premature Infants Treated with Surfactant and Budesonide through Computer-Assisted Chest X-ray Analysis | doi.org/10.3390/ diagnostics14020214 | *Diagnostics* 2024, *14*, 214. | <https://www.mdpi.com/2075-4418/14/2/214> | Not RCT  This study was not RCT and this study compared before and after received surfactant |
| Xu Y | 2024 | Therapeutic Efficacy of Budesonide Suspension Combined with Poractant Alfa Injection for Neonatal Respiratory Distress Syndrome and its Effect on Serum Ferritin and PAI-1 Expressions |  | Latin American Journal of Pharmacy - Volume 43, Issue 3, pp. 449-455 - published 2024-01-01. | <https://www.scopus.com/inward/record.uri?eid=2-s2.0-85191477077&partnerID=40&md5=9a25cdf9d336d8aa1daee5f94cf30d02> | Not RCT and irrelevant  This study was mentioned as RCT study, only divided to observed and control group. The results also were not relevant to our objective and outcomes. |
