# Supplementary material for: Efficacy of pulmonary surfactant with budesonide in premature infants: A systematic review and meta-analysis
Source: PLoS One. 2025 Jan 9;20(1):e0312561. doi: 10.1371/journal.pone.0312561 (PMC11717239; doi:10.1371/journal.pone.0312561)
Supplement: S1 Fig — (DOCX) [file pone.0312561.s006.docx]

**S2 Fig. Risk of bias summary of the included studies using the revised Cochrane risk of bias tool for randomized trials**

**
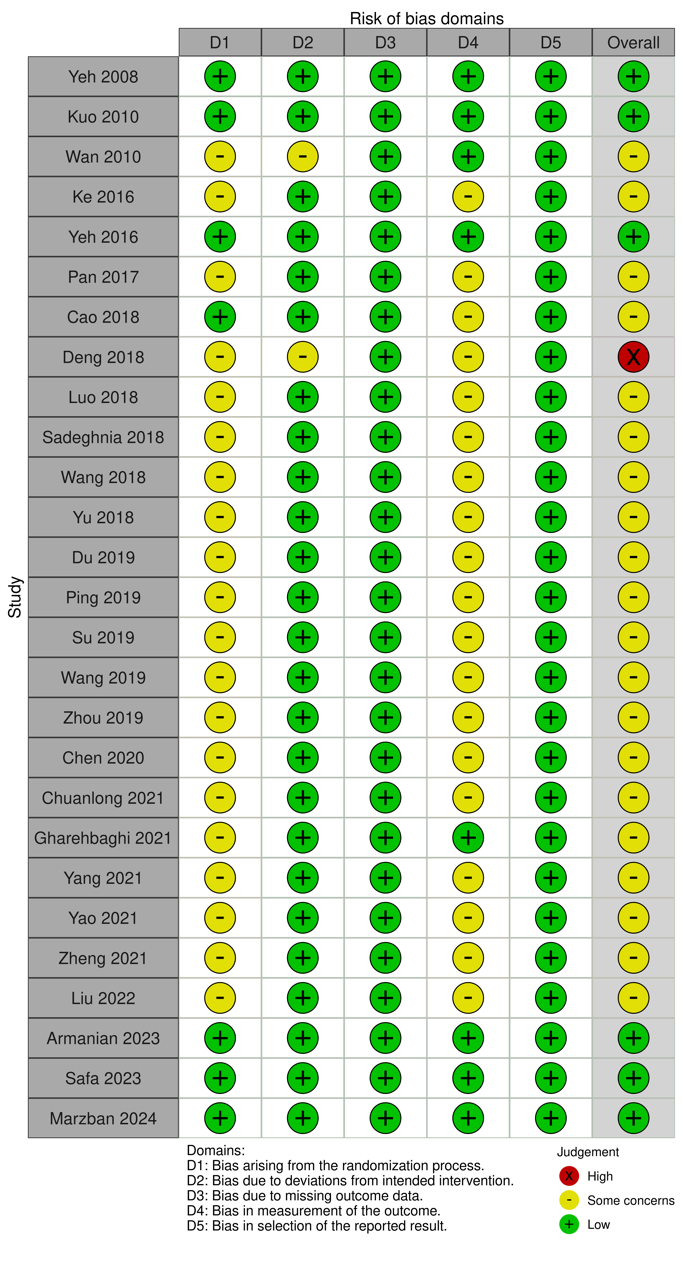
**
