# Supplementary material for: Efficacy of pulmonary surfactant with budesonide in premature infants: A systematic review and meta-analysis
Source: PLoS One. 2025 Jan 9;20(1):e0312561. doi: 10.1371/journal.pone.0312561 (PMC11717239; doi:10.1371/journal.pone.0312561)
Supplement: S2 Fig — (DOCX) [file pone.0312561.s007.docx]

**S6 Fig. Results of the outcomes in the systematic review and meta-analysis.** S(A) Ventilator-associated pneumonia (VAP) or respiratory infection. S(B) Pneumothorax. S(C) Pulmonary hemorrhage. S(D) Periventricular leukomalacia (PVL). S(E) Cerebral hemorrhage. S(F) Mental Development Index (MDI) score ≤ 69. S(G) Psychomotor Development Index (PDI) score ≤ 69. S(H) Mental Development Index (MDI) score. S(I) Psychomotor Development Index (PDI) score. S(J) Retinopathy of prematurity (ROP). S(K) Necrotizing Enterocolitis (NEC). S(L) Sepsis. S(M) Patent Ductus Arteriosus (PDA). BUD, budesonide; NB, nebulization; PS, pulmonary surfactant.

**S(A) Ventilator-associated pneumonia (VAP) or respiratory infection**

**S(B) Pneumothorax**

**S(C) Pulmonary hemorrhage**

**S(D) Periventricular leukomalacia (PVL)**

**S(E) Cerebral hemorrhage**

**S(F) Mental Development Index (MDI) score ≤ 69**

**S(G) Psychomotor Development Index (PDI) score ≤ 69**

**S(H) Mental Development Index (MDI) score**

**S(I) Psychomotor Development Index (PDI) score**

**S(J) Retinopathy of prematurity (ROP)**

**S(K) Necrotizing enterocolitis (NEC)**

**S(L) Sepsis**

**S(M) Patent Ductus Arteriosus (PDA)**
