# Supplementary material for: Efficacy of pulmonary surfactant with budesonide in premature infants: A systematic review and meta-analysis
Source: PLoS One. 2025 Jan 9;20(1):e0312561. doi: 10.1371/journal.pone.0312561 (PMC11717239; doi:10.1371/journal.pone.0312561)
Supplement: S3 Fig — (DOCX) [file pone.0312561.s008.docx]

**S7 Fig. Funnel plots.** (A)Incidence of bronchopulmonary dysplasia (BPD). (B) Redosing of pulmonary surfactant (PS). (C) Retinopathy of prematurity (ROP). (D) Sepsis. (E) Patent Ductus Arteriosus (PDA). (F) Duration of mechanical ventilation (MV) or invasive mechanical ventilation (IMV) (days). (G) Duration of oxygen supplementation (days). (H) Duration of hospitalization (days). BUD, budesonide; NB, nebulization; PS, pulmonary surfactant.

**A. Incidence of bronchopulmonary dysplasia (BPD)**

**B. Redosing of pulmonary surfactant (PS)**

**C. Retinopathy of prematurity (ROP)**

**D. Sepsis**

**E. Patent Ductus Arteriosus (PDA)**

**F. Duration mechanical ventilation or invasive mechanical ventilation (days)**

**G. Duration of oxygen supplementation (days)**

**H.** **Duration of hospitalization (days)**
